# Multiple *doublesex*-Related Genes Specify Critical Cell Fates in a *C. elegans* Male Neural Circuit

**DOI:** 10.1371/journal.pone.0026811

**Published:** 2011-11-01

**Authors:** Meagan S. Siehr, Pamela K. Koo, Amrita L. Sherlekar, Xuelin Bian, Meredith R. Bunkers, Renee M. Miller, Douglas S. Portman, Robyn Lints

**Affiliations:** 1 Department of Biology, Texas A & M University, College Station, Texas, United States of America; 2 Department of Biomedical Genetics, Center for Neural Development and Disease, University of Rochester, Rochester, New York, United States of America; Brown University, United States of America

## Abstract

**Background:**

In most animal species, males and females exhibit differences in behavior and morphology that relate to their respective roles in reproduction. DM (Doublesex/MAB-3) domain transcription factors are phylogenetically conserved regulators of sexual development. They are thought to establish sexual traits by sex-specifically modifying the activity of general developmental programs. However, there are few examples where the details of these interactions are known, particularly in the nervous system.

**Methodology/Principal Findings:**

In this study, we show that two *C. elegans* DM domain genes, *dmd-3* and *mab-23*, regulate sensory and muscle cell development in a male neural circuit required for mating. Using genetic approaches, we show that in the circuit sensory neurons, *dmd-3* and *mab-23* establish the correct pattern of dopaminergic (DA) and cholinergic (ACh) fate. We find that the ETS-domain transcription factor gene *ast-1*, a non-sex-specific, phylogenetically conserved activator of dopamine biosynthesis gene transcription, is broadly expressed in the circuit sensory neuron population. However, *dmd-3* and *mab-23* repress its activity in most cells, promoting ACh fate instead. A subset of neurons, preferentially exposed to a TGF-beta ligand, escape this repression because signal transduction pathway activity in these cells blocks *dmd-3/mab-23* function, allowing DA fate to be established. Through optogenetic and pharmacological approaches, we show that the sensory and muscle cell characteristics controlled by *dmd-3* and *mab-23* are crucial for circuit function.

**Conclusions/Significance:**

In the *C. elegans* male, DM domain genes *dmd-3* and *mab-23* regulate expression of cell sub-type characteristics that are critical for mating success. In particular, these factors limit the number of DA neurons in the male nervous system by sex-specifically regulating a phylogenetically conserved dopamine biosynthesis gene transcription factor. Homologous interactions between vertebrate counterparts could regulate sex differences in neuron sub-type populations in the brain.

## Introduction

DM (Doublesex/MAB-3) domain transcription factors represent one of the few regulators common to sexual development programs of the animal kingdom. Among the best characterized members of this family are the founding members *Drosophila melanogaster dsx (doublesex)*, *C. elegans mab-3* (*male abnormal-3*) and the vertebrate gene *Dmrt1* (*dsx-mab-3 related transcription factor 1*) [1], [2], [3]. The position of DM domain transcription factors in the sexual development gene hierarchy varies greatly across taxa. In some species, DM domain transcription factors are the primary sex determining signal, while in others they are dispatched sex-specifically in response to the primary signal to promote the sexual differentiation of discrete subsets of tissues [4]. A number of studies reveal that DM domain proteins promote sex-specific development by modifying the activity of general spatial and temporal patterning mechanisms, thereby ensuring that cells with the right characteristics arise in the correct axial location of the appropriate sex [5], [6], [7], [8], [9], [10], [11], [12]. Male/female differences in the nervous system are thought to underlie the complex, sex-specific behavioral repertoires associated with courtship and copulation. DM domain genes are expressed in the nervous systems of nematodes, fruit flies and even vertebrates [6], [13], [14], [15], [16], [17]. However, the developmental programs they modify and the sexual trait genes they control are largely unexplored.

The nematode worm *C. elegans* has two sexes: males and hermaphrodites, the latter being essentially females that can make their own sperm. Anatomical differences between the sexes have been described with single cell resolution through extensive cell lineage and electron microscopy studies [18], [19], [20], [21], [22], [23]. Males and hermaphrodites share in common many cells, of various types, and these are referred to as the core cells [24]. Although identical with respect to their lineal origin, some core cells in the two sexes may not be entirely equivalent due to the differential expression of terminal markers or differences in their connectivity, in the case of neurons [25], [26]. In addition to the core cells, males and hermaphrodites each have a complement of sex-specific cells that are generated only in one sex. In general, these sex-specific cells are produced from core blast cells that execute different lineages in each sex [19], [21]. This detailed knowledge of male and hermaphrodite anatomy provides an invaluable framework for investigating the neural basis for sex-specific behaviors and their evolution.

**Figure 1:**
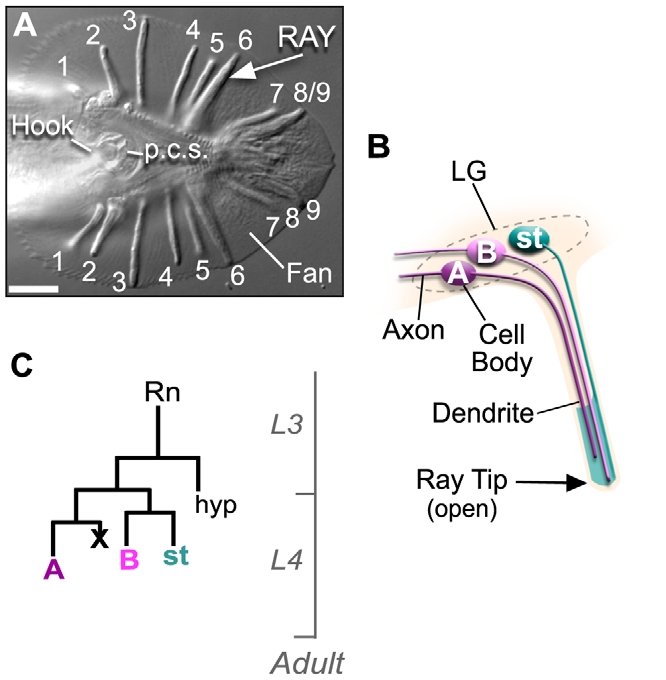
The ray sensilla are male-specific sensory organs required for mating. ***A.*** DIC micrograph of the male tail (ventral view, posterior to the right) showing the 9 bilateral ray pairs (numbered 1 to 9), the hook and post cloacal sensilla (p.c.s.). Magnification 1000×; Scale bar = 10 µM. ***B.*** Schematic showing the cell composition and organization of a single ray sensillum (longitudinal section). The ray contains the dendritic processes of the two ray neurons (type A and B) wrapped in the expanded process of a structural cell (st). The ray cell processes are wrapped in a layer of hypodermis and cuticle (not shown). In all rays, except the ray 6 pair, the ray tip opens to the environment. Ray cell bodies of the left and right side of the tail are located within the left and right lumbar ganglia, respectively (LG, dotted line). ***C.*** The ray sublineage program. The cells of a single ray derive from a common precursor cell, the Rn cell (where n stands for rays 1 to 9). The scale to the right indicates the timing of divisions relative to the developmental stages. At the end of the third larval stage (L3) nine Rn cells are present on each side of the tail. Rn cells divide by the stereotyped pattern shown to produce the three terminal cells of the ray (the A- and B-neurons and the structural cell), one hypodermal cell (hyp) and one cell that undergoes programmed cell death (x).

The *C. elegans* genome is predicted to encode eleven members of the DM domain gene family. *mab-3*, *mab-23* and *dmd-3(dm-domain gene-3)* are required for male development and have no obvious role in hermaphrodite sexual differentiation [6], [10], [13], [27]. *mab-3* and *mab-23* are expressed in several tissues in the male, including the male-specific sensory rays. These external sensilla are used by the male to sense and maintain physical contact with the hermaphrodite during mating [28]. There are nine bilateral pairs of rays (numbered 1 to 9) organized along the anterior-posterior axis of the tail and these are held together in an acellular fan (Fig. 1A). Each ray sensillum consists of the sensory endings of two ultra-structurally distinct sensory neurons, type A and B, wrapped in the process of a glial-like structural cell. These processes, in turn, are ensheathed in a layer of hypodermis and cuticle. Except for the ray 6 pair, the rays are open at their tips and the sensory ending of the B-neuron is exposed to the environment [19], [20](Fig. 1B). The A-neuron, B-neuron and structural cell of a single ray derive from a common precursor, the Rn cell (where n stands for rays 1 to 9), which executes a stereotyped pattern of cell division referred to as the ray sublineage (Fig. 1C). *mab-3* drives execution of the sublineage by promoting expression of the proneural gene *lin-32 (abnormal cell lineage-32)* in the Rn cell and its descendants [6], [10]. Although structurally simple, rays express complex combinations of characteristics in their constituent cells such that no two ray pairs are entirely identical. For example, the neurons of different ray pairs express different combinations of neurotransmitters and connect to different combinations of post-synaptic targets [19], [26], [29], [30]. *mab-23* regulates expression of neurotransmitter sub-type in a subset of ray neurons [13].

*dmd-3* is required for sex-specific remodeling of the tail during male sexual maturation [27]. *dmd-3* is also expressed in many other male tissues, including the ray sensory neurons and their ultimate targets, the body wall muscles. However, the role of *dmd-3* in the development of these cells and its relationship to *mab-3* and *mab-23* has not been investigated. Here, we show that *dmd-3* and *mab-23* regulate the expression of sub-type characteristics in the ray sensory neurons and in muscles. In the ray neurons, *dmd-3* and *mab-23* promote cholinergic (ACh) fate and limit dopaminergic (DA) fate by blocking the activity of *ast-1* (*axon steering defect-1*), which encodes an ETS-domain transcription factor required for dopamine biosynthesis gene transcription in all DA neurons [31]. In the muscles, *dmd-3* and *mab-23* regulate characteristics that render muscle cells responsive to upstream circuit activity. Through functional analyses, we show that these DM domain gene-regulated traits are critical for circuit output. Thus, interactions between DM domain transcription factors and a conserved, non-sex-specific patterning factor define circuit features that are essential for mating success.

## Results

### DM domain gene *dmd-3* regulates neurotransmitter fate in the male ray neurons

**Figure 2:**
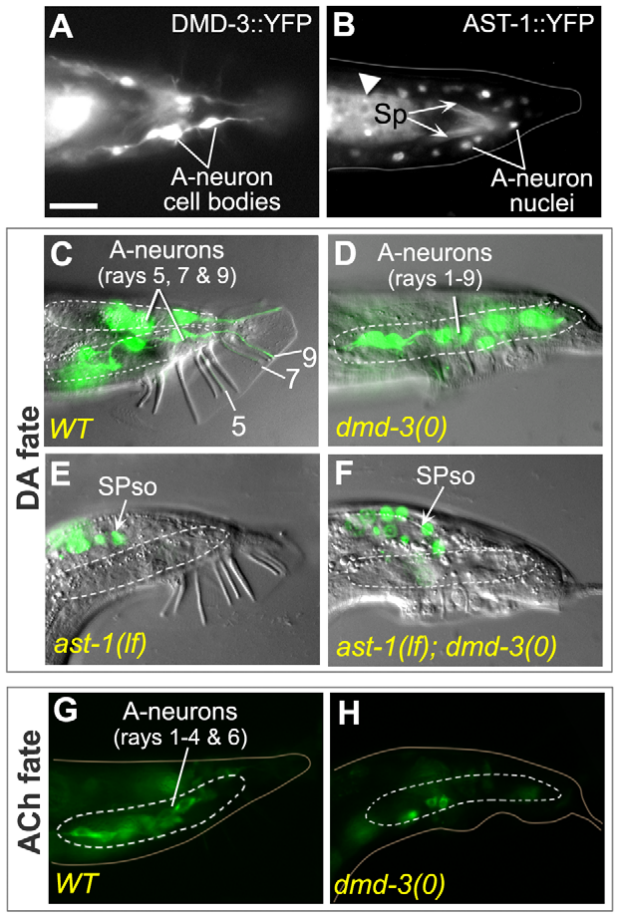
Interactions between *ast-1* and the DM domain genes regulate ray A-neuron neurotransmitter fates. ***A, B.*** *dmd-3* and *ast-1* are expressed in the A-neurons of the rays. Fluorescent micrographs showing DMD-3::YFP (***A***) and AST-1::YFP (***B***) expression in A-neurons of adult male rays (ventral view, posterior to the right, 600× magnification). The DMD-3::YFP fusion protein lacks a nuclear localization signal, consequently the fusion protein fills the entire neuron. The AST-1::YFP fusion protein contains the entire coding sequence and so localizes predominantly to cell nuclei [70]. Both reporters are expressed in the A-neurons of all rays. The arrowhead indicates gut auto-fluorescence; the arrows indicate copulatory spicule (Sp) auto-fluorescence. ***C–H.*** Expression of dopaminergic (DA) and cholinergic (ACh) fate in adult males of the genotype shown (lateral view, dorsal up, posterior to the right, 600× magnification). Ray cells of the left and right side of the tail are located in the left and right lumbar ganglia, respectively (white dotted line). ***C–F.*** DA fate expression. DIC and fluorescent micrographs of the same animal are superimposed. ***C*** **.** Wild type (*WT*) and ***D*** **.** *dmd-3(0)* males showing DA fate expression, visualized with a *pdat-1::GFP* reporter transgene. ***E.*** *ast-1(lf)* male showing the absence of DA marker expression in ray neurons. Here, the marker is CAT-2::GFP as *ast-1(lf)* mutations do not significantly affect *pdat-1::GFP* expression (See Table S1). In wild type males, CAT-2::GFP is expressed in the spicule socket cells (SPso) as well as the DA neurons (data not shown). *ast-1* mutations reduce, but do not eliminate SPso expression. ***F.*** *ast-1(lf); dmd-3(0)* male showing an absence of DA marker expression in all A-neurons (compare with ***D***). ***G, H.*** ACh fate expression. Fluorescent images of UNC-17::GFP expression in the A-neurons of ***G.*** *WT* and ***H.*** *dmd-3(0)* males. Scale bar (10 µM) applies to all images.

*dmd-3* was initially uncovered in a microarray study for ray-enriched transcripts [32]. Reporter transgenes subsequently revealed that *dmd-3* is expressed in the A-neurons of all rays [27] (Fig. 2A). *dmd-3* is also expressed in other tissues required for male reproductive behavior including the core muscles of the body wall and in a subset of male-specific muscles (see below). The ray A-neurons and the muscles correspond to the sensory and effector cells of the ray sensorimotor circuit, which controls male posture during mating [28]. Therefore, we asked whether *dmd-3* mutants have developmental defects in either of these cell types, beginning with the ray neurons.

The A-neurons of the rays stereotypically produce the neurotransmitters dopamine or acetylcholine [19], [28]. DA fate is defined by the expression of a battery of genes encoding factors required for dopamine biosynthesis and transport. These genes include the *C. elegans* orthologs of tyrosine hydroxylase (*cat-2*), aromatic amino acid decarboxylase (*bas-1*), GTP cyclohydrolase I (*cat-4*), the synaptic vesicular monoamine transporter (*cat-1*) and the dopamine re-uptake transporter *(dat-1)* [33], [34], [35], [36], [37], [38]. Of these, only *cat-2* and *dat-1* are specific markers of DA fate as *bas-1*, *cat-1* and *cat-4* are also expressed in other monoaminergic cell types. In the rays, *cat-2* and *dat-1* reporter expression is restricted to the DA A-neurons, which are located in ray pairs 5, 7 and 9 (Fig. 2C). The A-neurons of ray pairs 1 to 4 and 6, by contrast, adopt ACh fate and express genes specific to the production and transport of this transmitter, such as the acetylcholine vesicular transporter gene *unc-17* (Fig. 2G) or the cholinergic autoreceptor gene *gar-2* (Table S1) [28], [39], [40], [41].

**Figure 3:**
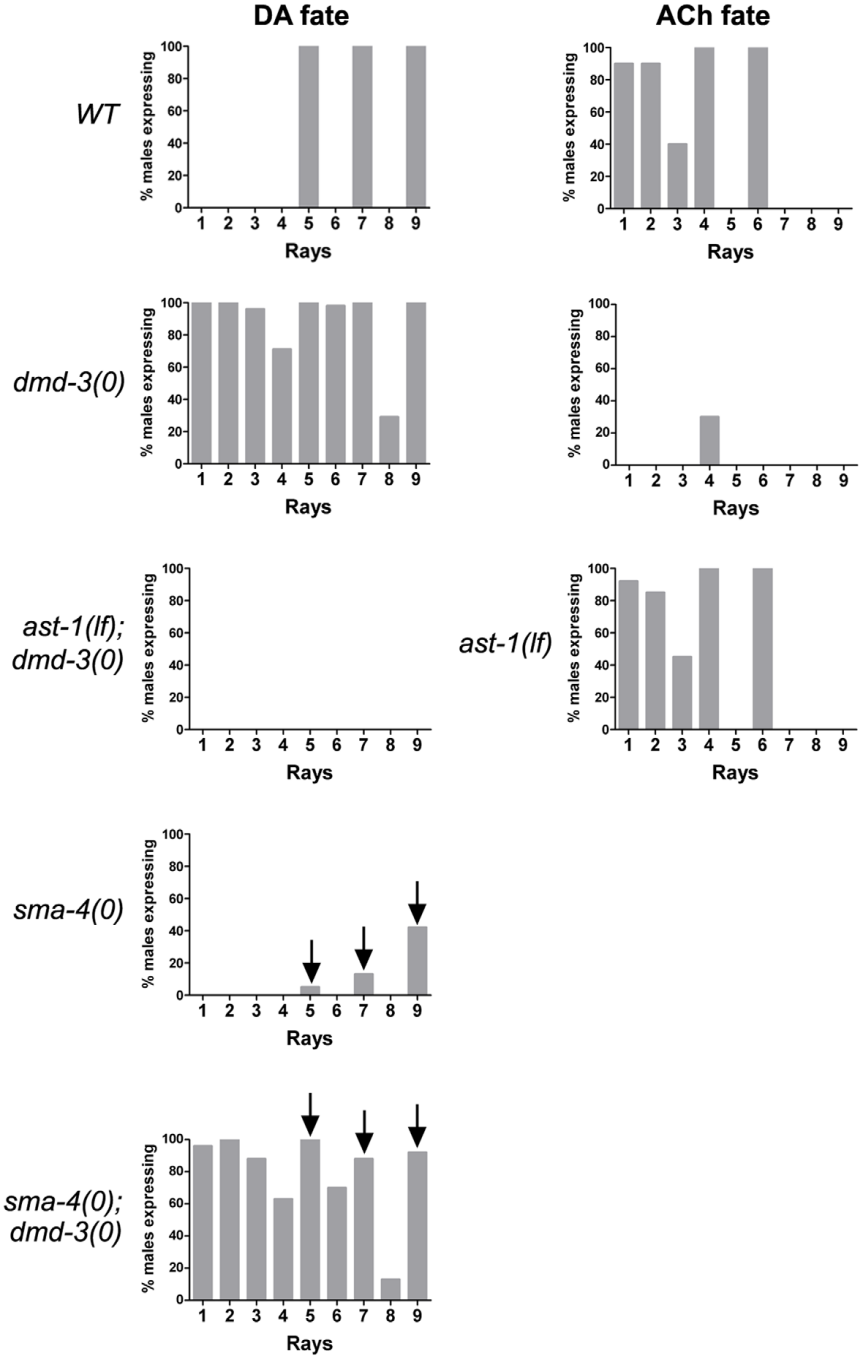
Quantification of ray neurotransmitter patterning defects in *dmd-3*, *ast-1* and DBL-1 pathway mutants. Populations of males of the genotype shown and carrying DA or ACh fate reporter transgenes were scored for the frequency of marker expression in rays indicated on the X-axis. The Y-axis shows the percentage of males that express the marker in a particular ray. n = 40–100 male tail sides scored (see Tables S1 and S2 for additional data). Except for *ast-1* mutants, the DA fate marker is *pdat-1::GFP*. For ACh fate, *unc-17::GFP* marker expression is shown. In wild type *(WT)* males, DA fate is restricted to the A-neurons of rays 5, 7 and 9 and ACh fate is expressed in the A-neurons of rays 1 to 4 and 6. In *dmd-3(0) males*, the A-neurons of additional rays variably express DA fate while ACh fate is almost completely eliminated. In *dmd-3(0) males*, the frequency of DA fate expression (visualized with CAT-2::GFP) is significantly reduced by *ast-1* loss of function. *ast-1* loss of function has no effect on ACh fate expression. *sma-4* encodes a SMAD of the DBL-1 signal transduction pathway that promotes rays 5, 7 and 9 identity. In *sma-4(0)* males, the A-neurons of these rays adopt DA fate at low frequency. Removal of *dmd-3* activity in a *sma-4(0)* background restores DA fate expression to 100% in these A-neurons (indicated by the arrows).

To determine if *dmd-3* activity is required for establishment of A-neuron neurotransmitter fates, we examined expression of DA and ACh fate markers in *dmd-3* null mutant males. In *dmd-3* males, we observed a dramatic alteration in the pattern of DA and ACh fates. In mutants, DA fate marker expression expanded to the A-neurons of most rays, while ACh marker expression was severely reduced (Fig. 2D, 2H, Fig. 3, Table S1). This loss of function phenotype reveals that in wild type males, *dmd-3* functions to suppress inappropriate expression of DA fate in the A-neurons and promote adoption of ACh fate. Not all aspects of A-neuron fate were affected by *dmd-3* loss of function. For example, expression of the TRP (Transient Receptor Potential) channel gene *trp-4* in A-neurons was unaffected by *dmd-3* loss of function (data not shown). The B-neurons do not express *dmd-3* suggesting that *dmd-3* is not required for their development. Consistent with this, we found no change in the expression of B-neuron markers in *dmd-3* mutants (the markers assessed were *flp-5*, *flp-17*, *tph-1*, *flp-11* and *pkd-2*; data not shown). Overall these results suggest that *dmd-3* is required specifically in the A-neurons where it regulates the choice between two neurotransmitter fates.

### *dmd-3* functions in parallel with *mab-23* to regulate ray A-neuron fate

The ray neuron patterning defects in *dmd-3* mutants are remarkably similar to that exhibited by *mab-23* mutants. Like *dmd-3*, *mab-23* is predicted to encode a DM domain transcription factor [13]. *mab-23* is also expressed specifically in the A-neurons, although only in the neurons of rays 1 to 4 and ray 6. As in *dmd-3* mutants, *mab-23* males inappropriately express DA fate in many A-neurons at the expense of ACh fate (Table S1). Furthermore, in the A-neurons of rays 5, 7 and 9, *mab-23* and *dmd-3* are negatively regulated by the DBL-1(DPP-BMP-Like-1) signal transduction pathway and this inhibition allows DA fate to be established in these cells (see below).

The similarity between *dmd-3* and *mab-23* phenotypes suggests that the two DM domain genes might function in a common genetic pathway; for example, one factor might be required to activate transcription of the other. *mab-23* expression precedes that of *dmd-3* in the ray sublineage. *mab-23* is first evident at the ray precursor cell (Rn) stage [13], whereas *dmd-3* expression is not apparent until the A-neurons are born, two cell divisions later. To test whether *mab-23* is required for *dmd-3* transcription, we examined *dmd-3* reporter expression in *mab-23* mutant males. We observed that *mab-23* loss of function had no effect on *dmd-3* reporter expression. Conversely, *mab-23* expression was unaffected by *dmd-3* loss of function (data not shown). *dmd-3* and *mab-23* therefore do not regulate each other's transcription.

To gain further insight into the relationship between the two genes, we compared the ray patterning defects in *dmd-3* and *mab-23* null single mutants with *dmd-3 mab-23* double mutants. Although their mutant phenotypes were grossly similar, *dmd-3* and *mab-23* single mutants exhibited differences in the frequency and/or intensity of marker expression in particular rays. In all instances, the consequence of *dmd-3* loss of function was greater than that of *mab-23* loss of function. For example, in *dmd-3* mutants ectopic expression of *dat-1::GFP* (DA fate) in ray 2 was consistently stronger and more frequent than its expression in ray 2 of *mab-23* mutants. Conversely, *unc-17::GFP* (ACh fate) was expressed at consistently lower levels and at lower frequency in ray 4 of *dmd-3* mutants compared to *mab-23* mutants (Table S1). *dmd-3 mab-23* double mutants were identical to the *dmd-3* single mutant in all aspects of ray patterning (Table S1).

Taken together, these data suggest that *dmd-3* and *mab-23* may function independently to regulate DA/ACh fate, with *dmd-3* playing the more critical role in these fate choices. However, our data do not rule out more complex scenarios in which the two genes might function in a linear pathway; for example, where MAB-23 contributes, either directly or indirectly, to the activity of DMD-3.

### Ray DA neuron fate depends on the non-sex-specific transcription factor gene *ast-1*

The *C. elegans* nervous system contains a relatively small number of DA neurons. In addition to the three bilateral pairs of DA neurons in the sensory rays, the male nervous system contains eight bilateral pairs of mechanosensory DA neurons that are also present in the hermaphrodite nervous system [19], [33]. *ast-1* encodes an ETS-domain transcription factor that is required for establishing and maintaining DA sub-type identity in all of these cells. *ast-1* is expressed in developing and differentiated DA neurons where it activates dopamine biosynthesis gene transcription through specific sequences in their gene promoters [31]. Significantly, the *ast-1* vertebrate homolog *Etv1* (*ETS translocation variant 1*) has a similar role in establishing DA phenotype in interneurons of the mouse olfactory bulb [31]. This remarkable example of functional homology between distantly related species suggests that some aspects of dopamine neuron development may be controlled by a phylogenetically conserved gene network.

We found that *ast-1* is expressed in the A-neurons of all rays, not just those that adopt DA fate (Fig. 2B). This prompted us to ask whether *ast-1* might also be required for establishing ACh fate in this population. We therefore examined ACh marker expression in *ast-1* hypomorphs and *ast-1(RNAi)* males. Reducing *ast-1* function using either approach resulted in a severe reduction of DA fate expression in the ray A-neurons, as expected [31] (Fig. 2E, Fig. 3, Table S1). By contrast, *ast-1* loss of function had no impact on expression of ACh fate in this population (Table S1). Thus, *ast-1* appears to be required only for DA fate in the rays.

### *dmd-3* and *mab-23* repress *ast-1*-dependent induction of DA fate

Only a subset of A-neurons that express *ast-1* adopt DA fate, namely the A-neurons of rays 5, 7 and 9. This suggests that in all other A-neurons, *ast-1* function must be repressed. *dmd-3* and *mab-23* may be responsible for this repression as their loss of function causes ectopic expression of DA fate in many A-neurons. If this hypothesis is correct, then these ectopic fates should be *ast-1*-dependent and eliminating *ast-1* function in these backgrounds should eliminate these fates. We found this to be the case: *ast-1* loss of function significantly reduced the frequency of ectopic DA fates in *dmd-3* and *mab-23* mutants (Fig. 2F, Fig. 3, Table S1). Thus in a wild type background, *dmd-3* and *mab-23* repress *ast-1* activity in most A-neurons. Given that the *ast-1* translational reporter is expressed in the A-neurons of all rays and that this pattern is unaffected by *dmd-3* or *mab-23* loss of function (data not shown), the DM domain transcription factors likely inhibit *ast-1* by means of a post-translational mechanism.

*dmd-3* and *mab-23* promote ACh fate while simultaneously repressing DA fate in the same cells. Potentially, *ast-1* could have an analogous, but reciprocal, effect on ACh and DA fate choices *i.e.*, *ast-1* might repress ACh fate in addition to promoting DA fate. If so, then reducing *ast-1* function in *dmd-3* or *mab-23* males should restore ACh fate expression to wild type levels. However, we found that this is not the case and that the frequency of ACh fate remained low in *dmd-3* mutants treated with *ast-1(RNAi)* (Table S1). These data suggest that *ast-1* has no role in ACh fate regulation and that it functions specifically in the establishment of DA fate.

### The DBL-1 pathway blocks *dmd-3* activity in the A-neurons of rays 5, 7 and 9

*dmd-3* does not inhibit *ast-1* function in all rays. In wild type males, the A-neurons of three ray pairs (rays 5, 7 and 9) express DA fate in an *ast-1*-dependent manner, despite *dmd-3* expression in these cells. What factors are responsible for antagonizing *dmd-3* in these cells? A likely candidate is the TGF-beta-related DBL-1 pathway, which is required for establishing ray identity characteristics in rays 5, 7 and 9 [42]. In the absence of DBL-1 pathway function, rays 5, 7 and 9 are each transformed to their respective anterior neighbor: ray 5 is transformed to ray 4, ray 7 to 6 and ray 9 to 8 [42], [43], [44], [45], [46]. Multiple aspects of ray identity are affected by these transformations including neurotransmitter fate [30], [36]. In particular, the frequency of DA fate expression in rays 5, 7 and 9 is reduced from 100% per ray in wild type males to 0–40% in DBL-1 pathway mutants [13], [36] (Fig. 3, Table S2). Potentially this reduction could be due to elevated *dmd-3* activity in the transformed neurons, triggered by removal of the DBL-1 pathway. According to our model, this increase in *dmd-3* activity would, in turn, result in repression of *ast-1* and consequently repression of DA fate. If so, then inactivation of *dmd-3* in a DBL-1 pathway mutant background should alleviate this repression and restore DA fate expression to 100% in rays 5, 7 and 9. We therefore constructed *dmd-3*; DBL-1 pathway double mutants and examined DA fate. As predicted, the frequency of DA fate in rays 5, 7 and 9 was restored to 100% in *dmd-3*; DBL-1 pathway double mutants (Fig. 3, Table S2). These data argue that the DBL-1 pathway inhibits *dmd-3* activity in the A-neurons of rays 5, 7 and 9. This inhibition allows *ast-1* to function in these neurons and establish DA fate. In rays that are not exposed to the DBL-1 ligand during development, *dmd-3* activity is not blocked, so DA fate is repressed and ACh fate is established instead.

### *dmd-3* and *mab-23* mutants are severely defective in male mating behavior

**Figure 4:**
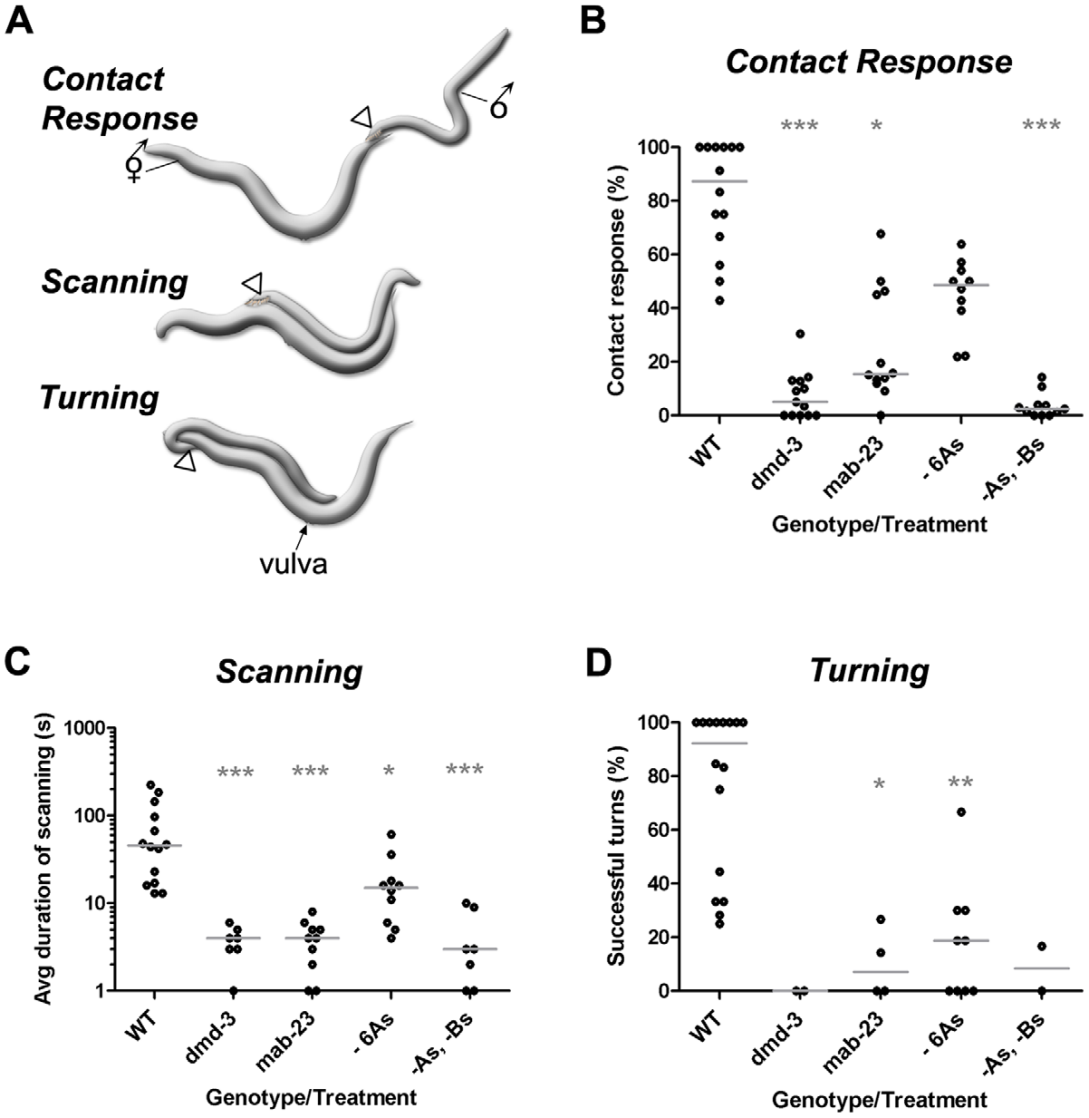
*dmd-3* and *mab-23* mutants have severe defects in male mating behavior. ***A.*** The first phase of male mating behavior involves a systematic search for the hermaphrodite vulva. This search requires apposition of the male tail (open triangle) against the hermaphrodite surface and is powered by backwards locomotion. Apposition behavior can be divided into three sub-behaviors: contact response, scanning and turning. Contact response requires both A- and B-neuron function, while scanning and turning are principally controlled by the A-neurons [28]. ***B–C.*** Quantification of mate apposition behavior in wild type (WT), *dmd-3* and *mab-23* null mutants. Each dot corresponds to the performance of a single male in a 15-minute mating assay (see Materials and Methods). The X-axis shows the genotype or treatment. “- 6As” corresponds to males lacking A-neurons in rays 1–6 due to their specific ablation with a laser micro-beam. The A-neurons affected by *mab-23* mutants are the same as those eliminated in the “- 6As” males. “-As, -Bs” corresponds to males lacking both A- and B-neurons in all rays due to their laser ablation [28]. The low sample size for mutant males or ray-neuron-ablated males in ***B*** and ***C*** is because fewer males in these categories progress as far as scanning or turning. Results of Nonparametric 1-way ANOVA (Kruskal-Wallis) are shown. Median, grey bars. Significance *** *p*<0.001; ** *p*<0.01; * *p*<0.05.

The rays are essential for sensing and maintaining contact with a hermaphrodite during mating [47]. Mating begins with initial recognition of a hermaphrodite through tail contact involving the rays (Fig. 4A). This induces the male to press (appose) his sensilla-studded tail against the hermaphrodite surface. The male then moves backwards along the hermaphrodite body, maintaining tail contact. Tail apposition brings the vulva-sensing organs of the ventral tail (the hook and post-cloacal sensilla, Fig. 1A) into direct contact with the hermaphrodite surface so that these sensilla can detect the vulva when the male passes over it and terminate the search. Apposition behavior can be divided into three identifiable sub-behaviors: contact response, scanning and turning (Fig. 4A). Contact response corresponds to the initial apposition of the tail and the commencement of backwards locomotion. As the male moves over the hermaphrodite surface, he effects the trajectory of the vulva search by altering his tail posture in response to unknown hermaphrodite surface cues. If the male's tail is far from the head or tail of the hermaphrodite, he adopts a “scanning” posture whereby his tail is pressed against the hermaphrodite and the posterior half of his body is parallel to hers. When the male approaches the hermaphrodite head or tail, he translocates himself to the other side of her body by making a deep ventral turn without losing tail contact. The male then resumes scanning along the other side of the hermaphrodite. The B-neurons appear essential only for contact response, whereas the A-neurons are critical for this sub-behavior as well as for scanning and turning [28]. As A-neuron patterning is disrupted in *dmd-3* and *mab-23* mutants, we investigated the impact of these fate transformations on mating behavior.

We assessed *dmd-3* and *mab-23* male mating behavior in standard mating assays. In these assays, we placed a single virgin, 1-day-old adult male with multiple adult, virgin hermaphrodites on a freshly prepared mating lawn, then digitally recorded their behavior for the first 15 minutes [28]. Wild type males usually exhibit contact response after the first tail contact with a hermaphrodite (Fig. 4B). Males then proceed to systematically search for the vulva without losing tail contact, driving the search with scanning and turning behavior (Fig. 4C, 4D). For wild type males, the average duration of scanning is often short (10–300 sec) because they usually identify the vulva the first or second time they pass over it (Fig. 4C). By contrast, *dmd-3* and *mab-23* males showed significant defects in apposition behavior. *dmd-3* males invariably ignored hermaphrodite contact (Fig. 4B). On the rare occasion where a *dmd-3* male did respond, he backed repeatedly against the hermaphrodite but could not firmly appose his tail against her. The mating behavior defects of *mab-23* males were less severe. *mab-23* males occasionally responded to contact and could press their tails against the hermaphrodite surface and back. However, their behavior was stochastic and males often lost contact shortly after initiating scanning or during turning (Fig. 4C, 4D). Consequently, the average duration of scanning for *mab-23* males was short and the frequency with which they completed turns without losing contact was low compared to control males.

### *dmd-3* and *mab-23* mutants respond abnormally to artificial stimulation of the ray neurons

**Figure 5:**
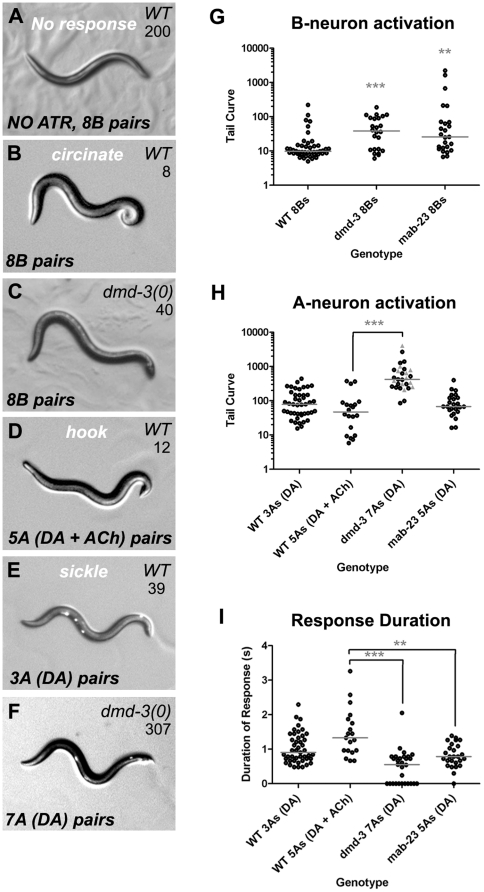
Ray neuron-induced tail-curl postures are abnormal in *dmd-3* and *mab-23* mutants. ***A–F.*** Tail postures generated by *ChR2-YFP*-expressing transgenic males after ChR2 activation with a blue light pulse (anterior left, posterior right). The number of ray neurons activated, their type and neurotransmitter fate (DA or ACh for the A-neurons) are indicated. The Tail Curve value for each male posture is indicated in the upper right of each image (see Materials and Methods for derivation). Except for ***A***, all males shown were grown in the presence of ChR2 co-factor all-*trans*-retinal (ATR). “Circinate”, “sickle” and “hook” refer to the shape of the tail posture. ***A***. A wild type *(WT)* transgenic male grown without ATR (NO ATR) shows no change in tail posture when pulsed with blue light because ChR2 is non-functional in the absence of ATR. In ***A–F***, the transgenes used were as follows: *ppkd-2::ChR2-YFP (* ***A–C*** *)*, *ptba-9::ChR2-YFP (* ***D*** *)*, *pdat-1::ChR2-YFP (* ***E*** *,* ***F*** *)*. The bright fluorescent spots in ***E*** and ***F*** correspond to expression of co-transformation marker UNC-122::GFP in coelomocytes. ***G, H.*** Quantification of Tail Curves generated by males after ChR2-mediated activation of ray neurons. Tail Curve values (Y-axis, log scale) were calculated for individual transgenic males expressing ChR2-YFP in the neurons indicated (X-axis) [28], (Materials and Methods). Each point corresponds to a single male: Black circles (responders): tail curl response with or without backing; Grey triangles (non-responders): no change in tail posture, forward locomotion uninterrupted. ***I.*** The duration of the ChR2-mediated A-neuron tail curl response for males shown in ***H*** (see Materials and Methods for derivation). In ***G–I*** the results of Nonparametric 1-way ANOVA (Kruskal-Wallis) are shown. Median, grey bars. Significance *** *p*<0.001; ** *p*<0.01; * *p*<0.05.

All mate apposition behaviors involve ventral deflection of the male tail to either establish or maintain tail contact with the hermaphrodite surface during the vulva search. This ventral bending can be induced in the absence of hermaphrodite contact, through the artificial stimulation of A- or B-neurons using light-inducible Channelrhodopsin (ChR2) from *Chlamydamonas reinhardtii* (Fig. 5) [28], [48]. This cation channel opens in response to blue light. When expressed heterologously in neurons, ChR2-mediated cation influx results in neuron depolarization. By genetically-targeting expression of ChR2 to different ray neuron types and sub-types, we can quantify the degree of ventral bending induced by their activation in wild type and mutant backgrounds [28]. To target expression of *YFP*-tagged *ChR2 (ChR2-YFP)* to the B-neurons, we placed this gene under the control of the *pkd-2* promoter (*ppkd-2*) [49]. To drive expression to the A-neurons, we used the *tba-9* promoter (*ptba-9*), which is expressed in almost all A-neurons [38], [50], or the *dat-1* promoter (*pdat-1*), which is expressed in the A-neurons of rays 5, 7 and 9 in wild type males or in most A-neurons in the DM domain gene mutants. To stimulate ChR2-expressing neurons, adult virgin transgenic males were exposed to a 400–700 msec pulse of blue light in the absence of hermaphrodites and their response was recorded. For each male, we derived a Tail Curve value for the posture they adopted, using a formula based on the radius of curvature [28] (Materials and Methods).

In wild type males, A- and B-neuron populations produce qualitatively different postures when artificially activated. The B-neurons induce a robust, tight ventral curl (circinate: a spiral with the tip innermost; typical Tail Curve values of 10–20; Fig. 5B, 5G). The A-neurons induce open ventral curls that fall into two main classes: a shallow curl (“sickle”; with Tail Curve values >30; Fig. 5E, 5H) and a deep curl (“hook”, Tail Curve values <30; Fig. 5D, 5H). Co-activation of A- and B-neurons, which emulates neuron activity during contact response, generates a posture that is intermediate to the sickle and circinate curl. These data suggests that both A- and B-neuron pathways contribute to the overall contact response posture. Potentially, the B-neurons, with their robust responsiveness, confer a rapidly-induced, downward force. A-neuron output may prevent over-curling, so that the ventral surface of the tail is more exposed and makes better contact with the hermaphrodite cuticle [28].

*dmd-3* and *mab-23* males exhibited abnormal response to both A- and B-neuron artificial stimulation. In both mutant backgrounds, B-neuron activation produced highly variable responses ranging from almost wild type to severely abnormal (Fig. 5C, 5G, Fig. S1C; Tail Curve values 30–1000). When the A-neurons were activated, we observed greater differences between the two mutant strains. When 5 or more A-neuron pairs are activated in *dmd-3* mutants, males either did not respond or produced an extremely weak curl that lasted for half the duration of the wild type response (Fig. 5F, 5H, 5I). *mab-23* males could produce a tail curl, however, this response was more comparable to activation of only 3 DA A-neuron pairs in a wild type background, rather than to 5 A-neurons pairs, both in terms of the type of posture generated (sickle only) and the duration of the response (Fig. 5H, 5I, Fig. S1E; Tail Curve values >30).

These experiments indicate that both A- and B-neuron-controlled neural pathways are affected in *dmd-3* and *mab-23* mutants. The B-neurons do not express *dmd-3* or *mab-23* and do not appear to require either gene for their development. However, as discussed below *dmd-3* and *mab-23* are expressed in muscle targets of the rays, thus abnormal response could be due to mis-specified muscles. Severe functional defects in both A- and B-neuron-controlled pathways would explain why *dmd-3* mutants are unable to mount a response to contact as both neural pathways are necessary for this sub-behavior. Indeed, *dmd-3* mutants are behaviorally identical to males in which all A- and B-neurons have been laser-ablated (the “-As, -Bs” treatment, Fig. 4B–D). In *mab-23* mutants, both pathways appear to be partially functional according to our ChR2 assay results. This would explain why *mab-23* males can execute each apposition sub-behavior, albeit with variable success. The stochastic nature of their behavioral defects suggests that sometimes this partial circuit activity is sufficient to mount a response but sometimes is not. This same stochastic behavior is exhibited by wild type males in which A-neurons in rays 1 to 6 have been ablated with a laser (the “- 6As” treatment, Fig. 4B–D), the same neurons affected by *mab-23* loss of function. However, *mab-23* males are typically worse than these “-6As” ablated males which suggests that the mutants harbor defects in other cells besides the A-neurons, such as the circuit muscles. While the ChR2 assays reveal that A- and B-neuron motor pathways are disrupted in the mutants, they do not tell us whether the defects lie in the sensory neurons, efferent targets or both. The experiments presented below attempt to address this question by assaying muscle function more directly.

### *dmd-3* and *mab-23* are expressed in muscles of the ray sensorimotor circuit

**Figure 6:**
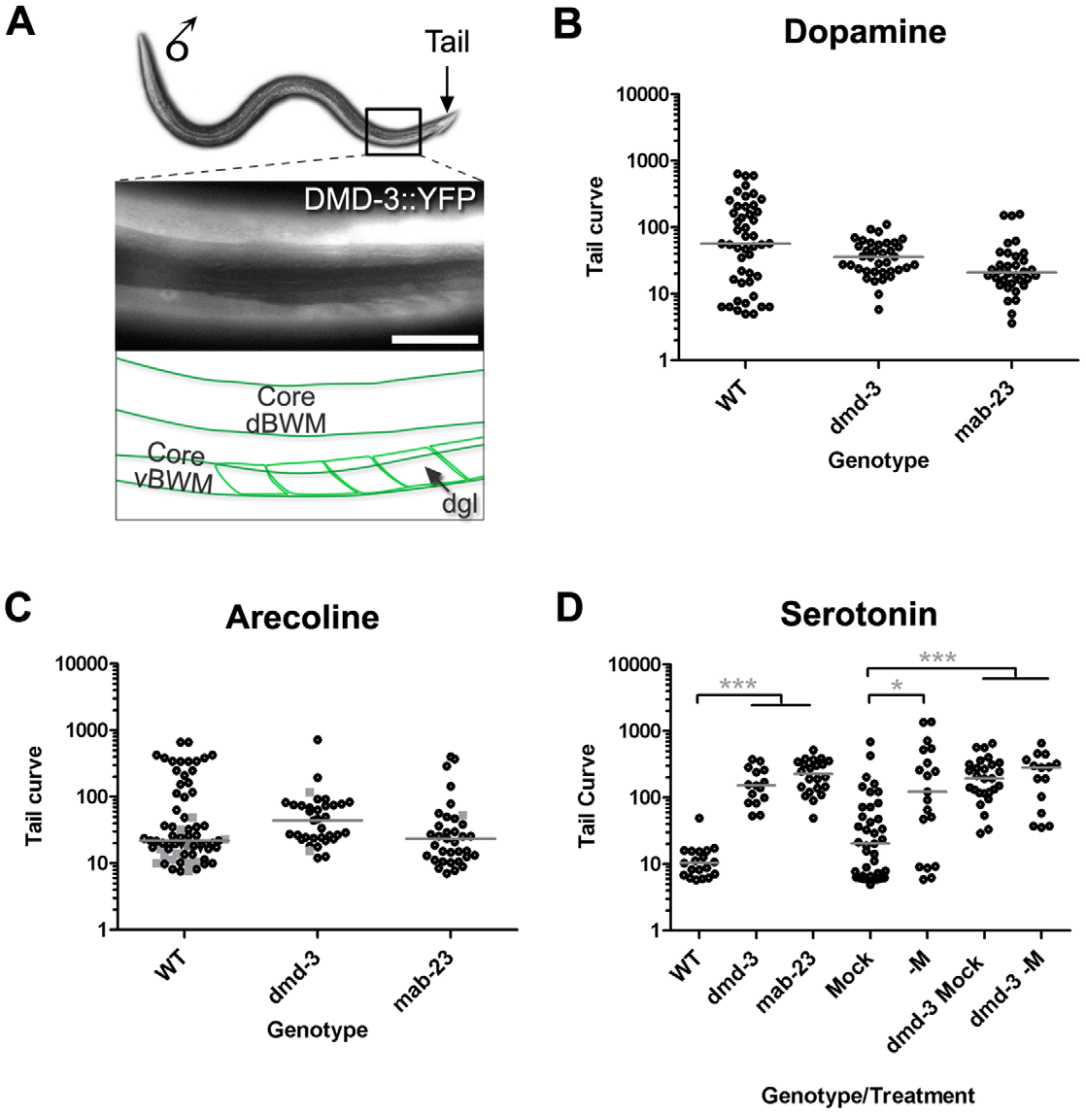
*dmd-3* and *mab-23* mutants have functionally abnormal muscles. ***A.*** DMD-3::YFP is expressed in core and male-specific muscles. A fluorescent micrograph showing DMD-3::YFP expression in muscles of the male posterior (lateral view, dorsal uppermost, posterior to the right). The region shown corresponds to the boxed area on the worm image above. DMD-3::YFP is expressed in dorsal and ventral core body wall muscles (dBWMs and vBWMs, respectively) and in the male-specific diagonal muscles (dgl, the arrow indicates a single dgl muscle). The dgl muscles lie immediately beneath the core vBWMs. Magnification: 600×. Scale Bar = 10 µM. ***B–D.*** Tail curves generated by adult males after exposure to exogenous neurotransmitter receptor agonists. Adult males of the genotype or treatment shown (X-axis) were incubated in solutions of ***B.*** 50 mM dopamine ***C.*** 1 mM arecoline ***D.*** 50 mM serotonin and their tail curls were measured (Y-axis log scale; see Materials and Methods). Each dot corresponds to the performance of a single male. In arecoline, wild type males can adopt a dorsal (grey squares) or ventral curl (black dots). In *dmd-3* and *mab-23* mutant populations, dorsal curls are infrequent, consequently the ratio of dorsal: ventral tail curls for the two populations is significantly different from that of wild type (*p*<0.001). However the form of the ventral curl posture produced by mutants is similar to that of wild type males (see Fig. S2). In ***D***, the “-M” treatment corresponds to males in which the M sex-muscle precursor cell has been ablated. The “Mock” treatment corresponds to males which were exposed to anesthetic but not subjected to laser ablation treatment. Results of Nonparametric 1-way ANOVA (Kruskal-Wallis) are shown. Median, grey bars. Significance *** *p*<0.001; ** *p*<0.01; * *p*<0.05.

*dmd-3* and *mab-23* are expressed in core body wall muscles and male-specific muscles of the tail [13] (Fig. 6A). Both muscle groups are necessary for ray-mediated control of tail posture during mating [28], [29], [51] and receive both direct and indirect inputs from the ray neurons [26]. The core body wall muscle system consists of 95 rhomboid-shaped muscle cells. These are tiled from the head to the tail in two dorsal and two ventral bundles. Males have an additional 41 male-specific muscles and these are located in the posterior [19], [20]. *dmd-3* is expressed in all core body wall muscles in both sexes and in 15 male-specific muscles called the diagonal muscles. These diagonally-oriented muscles attach at the lateral sides and the ventral midline in the posterior half of the animal (Fig. 6A). *mab-23* is also expressed in the core body wall muscles, but is limited to the posterior cells, as well as in the diagonal muscles [13]. Neither *dmd-3* nor *mab-23* males show obvious morphological defects in their muscles. However, in previous studies we observed that *mab-23* males respond poorly to exogenous serotonin (5HT), a neurotransmitter that induces ventral tail curling in wild type males, possibly by acting through receptors on the muscles. This suggests that in *mab-23* mutants, some muscles may be functionally defective [13]. We therefore performed pharmacological assays with additional agonists to assess muscle function in the mutants.

### *dmd-3* and *mab-23* mutants have defects in muscles of the ray sensorimotor circuit

When wild type adult males are exposed to exogenous ACh or DA receptor agonists, they curl their tails ventrally, a response similar to that produced by ChR2-mediated ray neuron activation [51] (this study). A number of ACh and DA receptor genes are expressed in core and male-specific muscles of the tail, suggesting that agonist-induced tail curling could, at least in part, involve activation of muscle-expressed receptors [51], [52] (data not shown). Thus testing the response of mutant males to receptor agonists allows us to assess directly the functionality of circuit muscles, bypassing potential impediments in ray neuron function.

Wild type males respond to exogenous dopamine by curling their tails ventrally, to varying degrees, and becoming paralyzed (Fig. 6B, Fig. S2A). *dmd-3* and *mab-23* males also responded to dopamine by curling their tails ventrally except that the curl was deeper (Fig. 6B, Fig. S2B, S2C). As noted above, *dmd-3* males produced little to no response when the A-neurons were artificially activated using ChR2. The fact that mutants can respond to exogenous dopamine argues that the muscles in *dmd-3* mutants are functional and can respond to this transmitter. This suggests that the failure of *dmd-3* males to respond to ray neuron stimulation is due to a defect in the ray neurons themselves. Similarly, the relatively weak output of ChR2-activated A-neurons in *mab-23* mutants also suggests a sensory, rather than a muscle cell defect.

When wild type males are exposed to ACh receptor agonist arecoline, they initially curl their tails dorsally then gradually make the transition to a ventral curl, such that after 5 minutes the ratio of dorsal to ventral curling in the population is 1∶4 (Fig. 6C, where the grey squares correspond to dorsal curls and the black dots to ventral curls; Fig. S2D, S2E). Although tail apposition during mating involves predominantly ventral muscle contraction, dorsal muscle activity is also thought to contribute and is regulated by cholinergic signaling [51]. In arecoline, the DM domain gene mutants produced ventral tail curls similar to wild type males, but rarely curled their tails dorsally. Consequently, the ratio of dorsal to ventral tail curls in *dmd-3* and *mab-23* male populations is 1∶35 and 1∶17, respectively (Fig. 6C, Fig. S2F, S2G). As core dorsal body wall muscles are necessary for dorsal curling in ACh receptor agonists [51], these data argue that the dorsal core muscles are defective in the mutants. Thus, in addition to having fewer ray neurons that synthesize acetylcholine, a subset of muscles in the DM domain gene mutants are defective in their ability to respond to this transmitter.

Serotonin (5HT) has been implicated in control of tail posture during mating [28], [29], [53]. In the male nervous system, 5HT is synthesized by the B-neurons of rays 1, 3 and 9, the ventral nerve cord motor neurons CP1 to CP6 and an unidentified, male-specific neuron of the pre-anal ganglion [19], [26], [29], [30]. In *dmd-3* and *mab-23* males, the fates of these cells are unaffected (data not shown). Motor neurons CP1 to CP6 synapse directly onto ventral core and male-specific muscles of the tail, including the diagonal muscles [26]. This connectivity suggests that CP1 to CP6 could effect tail apposition through the direct activation of these muscles. Exposure of wild type males to exogenous 5HT induces tail ventral curling (Fig. 6D, Fig. S2H). By contrast, *dmd-3* and *mab-23* males could not curl their tails in 5HT (Fig. 6D, Fig. S2I, S2J). Thus, 5HT-dependent control of tail muscles is severely disrupted in the DM domain gene mutants. To test whether 5HT-induced tail curling in wild type males depends on core or male-specific muscles, we ablated all male-specific muscles in wild type males and tested their response to 5HT. We eliminated all male-specific muscles by ablating their common precursor M, present in the first larval stage. Wild type adult males that lack all male-specific muscles (“-M” treatment, Fig. 6D) could still curl their tails and could even produce the tight ventral curls exhibited by un-operated males (“Mock” treatment, tight tail curls have values <10, Fig. 6D). This suggests that, while male-specific muscles contribute to tail curling, core muscle activity plays a significant role and can even compensate for the absence of male-specific muscles. In contrast to wild type males, *dmd-3* mutants lacking male-specific muscles (“*dmd-3* -M”, Fig. 6D) were incapable of tail ventral curling. These data indicate that the core muscles in *dmd-3* mutants are defective in their ability to respond to 5HT.

Overall, these agonist studies suggest that *dmd-3* and *mab-23* have functional defects in cells downstream to the sensory rays, most likely the muscle cells as these are the only circuit cells that express *dmd-3* and *mab-23* besides the ray neurons. Taken together, the data argue that *dmd-3* and *mab-23* males have both sensory and efferent cell defects and these are sufficient to account for the poor mating efficiency of the mutants.

## Discussion

**Figure 7:**
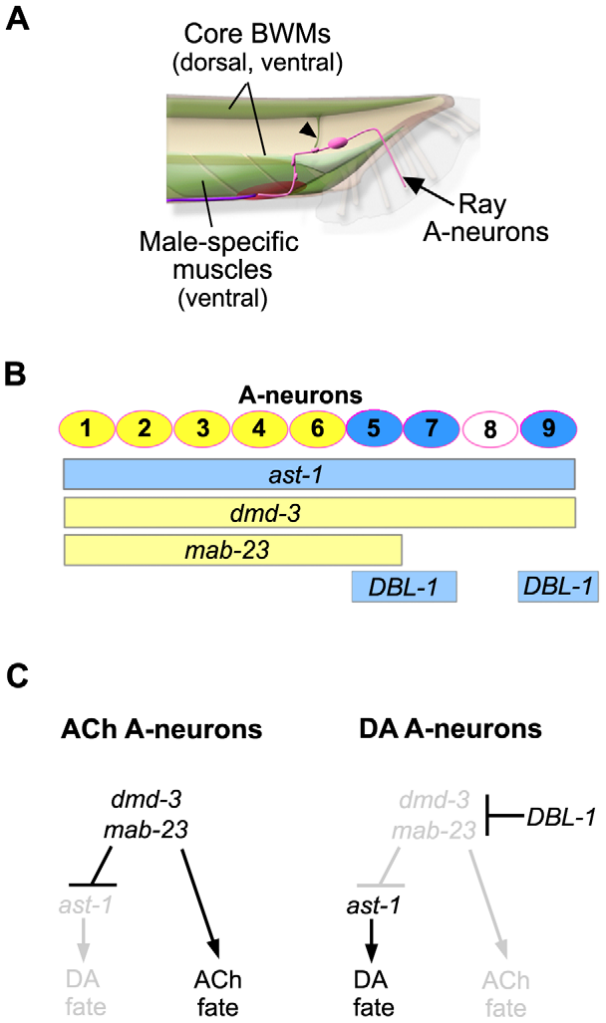
*dmd-3* and *mab-23* specify multiple cell fates in the ray sensorimotor circuit. ***A.*** Schematic of the male tail (lateral view, posterior right) indicating those tissues that express *dmd-3* and *mab-23* reporters. In the A-neurons of the rays (pink), *mab-23* and *dmd-3* regulate DA and ACh fates. In the muscles (green), *dmd-3* and *mab-23* regulate unknown characteristics that render the muscles responsive to ray circuit control. Ray axons synapse directly on to muscles of the tail *en route* to the ventral pre-anal ganglion (red oval). Synapses with dorsal muscles occur on muscle arms that project ventrally (arrowhead). The rays also make indirect connections with muscles through inter- and motor neurons of the pre-anal ganglion and ventral nerve cord (purple line). ***B.*** Sites of action for *ast-1*, *dmd-3*, *mab-23* and the DBL-1 pathway in the A-neuron population. The A-neurons (circles) of rays 1 to 9 stereotypically express ACh (yellow) or DA (blue) fate. The bars below indicate the zone of activity for a specific regulator within the A-neuron population. *ast-1* and the DBL-1 pathway (blue bars) promote DA fate while *dmd-3* and *mab-23* (yellow bars) repress DA fate and promote ACh fate. ***C.*** A genetic model for control of A-neuron neurotransmitter fate. In the A-neurons of rays 1 to 4 and ray 6, *dmd-3* and *mab-23* activity promote adoption of ACh fate and simultaneously suppress DA fate, which is conferred by *ast-1*. The A-neurons of rays 5, 7 and 9 are differentially exposed to the DBL-1 ligand during development. Activation of the DBL-1 signal transduction pathway within the A-neurons of these rays blocks *dmd-3* and *mab-23* activity. Consequently, ACh fate is not expressed and *ast-1* can activate transcription of dopamine terminal fate genes.

How biological sex is superimposed on the developing nervous system is a broadly relevant question that impacts the genetic basis of sex-specific behaviors, gender-bias in human disease states and mechanisms of species evolution. Developmental studies reveal that sex chromosome signals or their downstream effectors modulate axial patterning programs to generate male/female differences in animal body plans. However, there are few examples, particularly in the nervous system, where the identity of these developmental regulators and the sexual trait genes they control are known [8], [11], [54]. Here we show that sexual regulators of the *C. elegans* DM domain gene family repress the activity of a non-sex-specific, DA fate specification factor, thereby limiting the number of dopamine neurons in the male nervous system. Loss-of-function mutations in several ray development genes cause expanded expression of DA fate among the A-neurons suggesting that all A-neurons are programmed with the potential to adopt this fate [13], [30], [55]. In this study we show that this potential depends on *ast-1*, which is essential for DA neuron fate elsewhere in the worm [31], [56]. In the rays, *dmd-3* and *mab-23* repress *ast-1*-dependent DA fates and promote ACh fate instead. In the A-neurons of rays 5, 7 and 9, however, their activity is blocked by the DBL-1 pathway and this counter-repression allows DA fate to be established in these cells (Fig. 7). The DM domain genes and *ast-1* appear to act instructively in the ACh/DA fate decision. *ast-1*, although expressed in all A-neurons, appears to act specifically in establishment of DA fate. *dmd-3* and *mab-23* function in both the ACh and the DA fate choice, but with apparently opposite roles in each (Fig. 7). Similar bi-functionality has been described for other DM domain transcription factors [6], [11], [17]. However, it should be noted that apparent transcriptional activation is sometimes the indirect consequence of repression. For example, transcriptional activation of *lin-32* in the ray sublineage by *mab-3* is due to *mab-3*-mediated repression of *ref-1(regulator of fusion-1)*, a *Hes*-related neurogenic gene that otherwise suppresses *lin-32* expression [10].

*dmd-3*, *mab-23* and *ast-1* are predicted to encode transcription factors and all are expressed in developing and differentiated A-neurons (Fig. 7). It is therefore possible that their interaction occurs at the promoters of the biosynthetic genes that confer the ACh and DA neuron phenotype. Indeed, *ast-1* has been shown to directly control DA biosynthesis gene expression through specific DNA sequences in the promoters of these genes [31]. A consensus DNA recognition sequence has also been identified for DMD-3 (M. Murphy, D. Zarkower, D.S. P., unpublished) and this resembles the binding site for MAB-3 [57]. Although we identified potential DMD-3 binding sites in some neurotransmitter biosynthesis gene promoters, mutagenesis of these sites failed to alter gene expression (data not shown). This suggests that DMD-3 may function through other binding sites in these promoters. Possibly DMD-3 binds as a heterodimer or in a complex, in which case its site-specificity may be different [58], [59]. Alternatively, DMD-3 could activate other transcription factors that, in turn, directly control neurotransmitter biosynthesis gene transcription.

The A-neuron patterning defects in *dmd-3* and *mab-23* males have a deleterious impact on ray sensorimotor circuit function. In *dmd-3* mutants, almost all A-neuron neurotransmitter fates are altered and the population is essentially incapable of producing a motor response when artificially stimulated with ChR2. In *mab-23* mutants, fewer A-neurons are mis-specified, however the remaining functional neurons cannot compensate for this deficit. The extra dopamine produced by the transformed A-neurons in *dmd-3* and *mab-23* mutants should be able to induce ventral bending of the tail, based on our pharmacological assays. However, the fact that it does not, particularly in *dmd-3* males, suggests that the A-neurons in these animals are not functional. Possibly, the fate transformation caused by *dmd-3* and *mab-23* loss of function is incomplete and affected neurons may lack a critical component necessary for dopamine release. *dmd-3* and *mab-23* are also expressed in core and male-specific muscles of the circuit, during their differentiation and in the adult. The results of both the arecoline and serotonin tail curl assays provide clear evidence that *dmd-3* and *mab-23* are required in at least the core muscles. *dmd-3* and *mab-23* males were more defective in their response to serotonin than to dopamine or arecoline, which suggests that their muscle defects are limited to expression of certain sub-type characteristics. The response to all agonists was similar for the two mutant strains, which suggests that *dmd-3* and *mab-23* may regulate expression of common characteristics in muscles, as they do in the rays.

To what extent sex-specific behaviors are the product of sex-specific versus sex-common circuitry has important implications for understanding how sex-specific behaviors evolve and the mechanism underlying gender-bias in human disease states. In *C. elegans* and *Drosophila melanogaster* detailed wiring diagrams of male and female nervous systems provide insight into this general question. In *Drosophila melanogaster*, high-resolution analyses of *fruitless* and *dsx* expression patterns reveal circuits composed of male/female common cell types that are sex-specifically modified as well as sex-specifically generated cell types [15], [16], [60], [61], [62]. In *C. elegans* wiring diagrams for the entire hermaphrodite nervous system and for part of the male have been generated through electron micrograph serial section reconstructions [21], [22], [26]. In the male it is possible to trace multiple neural pathways from the ray neurons to their muscles targets and these pathways are composed of both core and male-specific cell types [26]. The robustness and acuity of apposition behavior suggest that processing is distributed through these multiple pathways. Potentially, the ray-to-muscle direct connections correspond to the primordial circuit upon which the extant network is based. *dmd-3-* and *mab-23-*dependent cells types account for only a fraction of the cells in the network. The factors controlling the development of other male-specific cell types or that drive sex-specific modification of certain core neurons are currently unknown.

In mammals, the central dogma of brain development poses that sex steroids from the gonads organize neural circuits in the developing brain [63]. However, recent evidence suggests that non-gonadal factors also play a role [64], [65]. HMG-box transcription factor SRY (Sex determining Region on Y) represents one such factor and has been shown to positively regulate dopamine neuron number in the male substantia nigra by controlling tyrosine hydroxylase gene expression [66]. DM domain gene family members are expressed in the vertebrate nervous system and could represent another mechanism for gonadal hormone-independent control [14], [67], [68]. For example, vertebrate gene *Dmrt4* is expressed in mouse olfactory sensory neurons and *Dmrt4* mutant males exhibit increased preference for other males, although the molecular basis of this defect is unclear [14]. In this study we show that DM domain genes in the nematode regulate a non-sex-specific transcription factor required for dopamine biosynthesis gene expression [31]. Given that both the sexual regulators and the DA fate effector are conserved, it is possible that similar interactions between their vertebrate counterparts could create sexually dimorphic differences in neuron populations in the brain.

## Materials and Methods

### Strains

The strains used in this study are as follows: *egIs1(pdat-1::GFP)* [69], *fsIs2(dmd-3::YFP+unc-122::GFP)*, *fsEx5(pgar-2::YFP+pha-1(+))*, *bxEx106, −107(unc-17::GFP+pha-1(+))* [41], *hdIs42(ast-1::YFP+rol-6(su1006))* [70], *fkIs3(ppkd-2::ChR2-YFP+ttx-3::mCherry)*, *fkIs1(pdat-1::ChR2-YFP+unc-122::GFP)*, *ast-1(rh300)II* [71], *sma-4(e729)III* [72], *unc-64(e246)III* [72], *pha-1(e2123ts)III* [73], *dmd-3(ok1327) and -(tm2863)V* [27], *mab-23(bx118)*, *-(e2518) and -(gk644)V*, *fkIs4V (ptba-9::ChR2-YFP+unc-122::GFP)* [28], *bxIs16V(cat-2::YFP+tph-1::CFP+pha-1(+))* [55], *him-5(e1490)V*, *lin-15B(n765ts)X* [74]. All strains used in this study carried the *him-5(e1490)* mutation which generates a *h*igh *i*ncidence of *m*ales [75]. Except for *pha-1(e2123ts)*, all strains were maintained at 20°C and cultured using standard conditions [72].

### RNAi experiments

*ast-1* and control (*pseudogene* C06C3.5) RNAi experiments (Table S1) were carried out as described in Ahringer (2006) [76]. Bacterial cultures containing the relevant dsRNA clones [77] were grown overnight in LB media containing 25 µg/mL of carbenicillin. Forty µLs of each culture was spread on NGM media that was supplemented with 1 mM IPTG and 25 µg/mL carbenicillin. Transgenic hermaphrodites carrying the relevant reporter genes were allowed to lay eggs on plates for approximately 24 hours. To enhance the sensitivity of neurons to RNAi, all transgenic reporter strains carried the *lin-15(n765ts)* mutation [74], [78]. F1 adult male progeny were mounted on 5% agarose pads containing anesthetic, then scored for the frequency of marker-positive ray neurons. Scoring was performed at 600× or 1000× magnification on a Zeiss D1 microscope equipped with epifluorescence. Fluorescent and Differential Interference Contrast (DIC) images were captured using a Zeiss MR Digital Camera and Zeiss Axiovision software (release 4.7).

### Laser-mediated M precursor ablations

The sex-specific muscle precursor M was eliminated in L1 wild type and *dmd-3* mutant males using standard laser ablation procedures [79]. After reaching the L3–L4 stage males were transferred to fresh plates to mature overnight in the absence of hermaphrodites before being subjected to exogenous 5HT assays.

### Mating behavior assays

Mating assays were carried out based on procedures described in Liu et al. (2007) [80]. Each assay was digitally recorded using a Zeiss AxioCam HS digital camera and AxioVision software (release 4.7). Trials ended after 15 minutes or after the male ejaculated, whichever occurred first. Execution of a specific sub-step in the behavior was counted using an Excel macro that logs keystrokes and timestamps [80]. Basic measures of mating behavior - contact response success, scanning duration and turn completion rate - were scored as follows. Contact Response: male tail contact with the hermaphrodite that resulted in placement of the tail against her surface and backing was scored as contact response. Other types of response that were not counted as a canonical contact response were attempted tail placement without backing, backing without tail placement or no response. Contact Response (%): [the number of contact responses/total number of tail contacts]×100. Average Duration of Scanning (sec): [the total time the male had his tail in contact with the hermaphrodite during scanning/total number of contact response events]. Completed Turns (%): [the number of turns completed/the total number of turning attempts]×100. Completed turns include sloppy or good turns followed by resumption of scanning. Turning types were classified as per Loer and Kenyon (1993).

### Channelrhodopsin-2 assays

All strains used for channelrhodopsin (ChR2) assays were maintained on plates spread with OP50 *E. coli* containing 50 µM all-*trans*-retinal (ATR) and, except during animal transfer or assays, were kept wrapped in foil. Generation of a functional ChR2 protein requires incorporation of a retinal moiety. *C. elegans* does not synthesize retinal, so all-*trans*-retinal (ATR) is provided in their bacterial food. Twenty-four hours before assaying, five early L4 males were placed on a plate freshly spread with 50 µM ATR in OP50. Assays were performed on a Zeiss M2 Imager stereomicroscope equipped with epi-fluorescence. Individual animals were pulsed for 500–700 msec with blue light (470/40 nm wavelength) while moving forward. Assays were recorded using a Zeiss AxioCam HS camera and AxioVision software. *ptba-9::ChR2-YFP* (*fkIs4*) males were used to assess the consequence of activating 5 or more A-neurons in wild type males. To assess the activity of the same neurons in DM domain mutants we used the *fkIs1* array in which ChR2 is under control of the *dat-1* promoter and is expressed in a similar set of A-neurons to the *ptba-9::ChR2-YFP* transgene in a wild type background. To activate the ray B-neurons males carrying the *ppkd-2::ChR2* transgene (*fkIs3*) were used. Tail Curve values were derived using Axiovision software tools as described in Koo *et al.* (2011) [28]. The frame in which maximum posture response is observed was extracted from the movie. A tail curl value was derived from this frame using a formula based on the radius of curvature of the curl. For non-responders, Tail Curve values correspond to the curvature of the tail during normal forward sinusoidal locomotion at the time interval after the pulse a response would normally be observed [28].

To calculate the duration of the tail curl postures measured in Fig. 5, the digital movie frames showing the beginning and end of the response were identified. Response Duration (sec): response end time – start time.

### Pharmacological Assays

Virgin, 1-day-old adult males were placed in a 100 µL drop of 50 mM 5HT (Sigma), 1 mM arecoline (Acros Organics) or 50 mM dopamine (Sigma) in a three-well glass dish. Serotonin and arecoline solutions were made in water while dopamine solutions were made in 2 mM acetic acid. When males adopted the maximum degree of tail curling (after 2 mins in 5HT or 5 mins in dopamine or arecoline, respectively) an image of the animal's posture was taken using a high-speed camera. A Tail Curve value was obtained for each male as described for the ChR2 assays above. Control males were placed in water (for serotonin and arecoline assays) or in 2 mM acetic acid (dopamine assays).

### Statistical Analysis

All statistical analyses were performed using GraphPad Prism.

## Supporting Information

Figure S1 **Ray neuron-induced tail-curl postures are abnormal in** ***mab-23*** **mutants.** Relates to Fig. 5. Images of tail postures generated by *ChR2-YFP* transgenic males after ChR2 activation with a blue light pulse (anterior left, posterior right). The number of ray neurons activated, their type (A or B) and neurotransmitter fate (DA or ACh for the A-neurons) are indicated. The Tail Curve value for each male posture is indicated in the upper right of each image (see Materials and Methods for derivation). Except for ***A***, all males shown were grown in the presence of ChR2 co-factor all-*trans*-retinal (ATR). “Circinate”, “sickle” and “hook” refer to the shape of the tail posture. ***A***. A wild type *(WT)* transgenic male grown without ATR (NO ATR) shows no change in tail posture when pulsed with blue light because ChR2 is non-functional in the absence of ATR. The transgenes used were as follows: ***A–C***. *ppkd-2::ChR2-YFP*. ***D.*** *ptba-9::ChR2-YFP*. ***E***, ***F.*** *pdat-1::ChR2-YFP*. The bright fluorescent spots in ***E*** and ***F*** correspond to expression of co-transformation marker UNC-122::GFP in coelomocytes.
(TIF)

Figure S2 **DM domain gene mutants show abnormal responses to exogenous neurotransmitter receptor agonists.** Relates to Fig. 6. Images of wild type (*WT*), *dmd-3(0)* and *mab-23(0)* males after exposure to the neurotransmitter receptor agonist indicated (anterior left and posterior right, see Materials and Methods). Tail Curve values (top right) were derived as described for ChR2 assays. ***D*** shows a WT type male curling its tail dorsally when exposed to arecoline. *dmd-3* and *mab-23* males rarely curl their tails dorsally in this agonist.
(TIF)

Table S1 ***dmd-3*** **,** ***mab-23*** **and** ***ast-1*** **regulate DA/ACh fate choice in the ray A-neurons.** Relates to Fig. 3. Expression of A-neuron fates in the genetic backgrounds indicated. The percentage of males that express the marker indicated in a particular ray is shown. Typically one side per male was scored. Dopaminergic (DA) fate: *cat-2* or *dat-1* reporters [36], [69], cholinergic (ACh) fate: *unc-17* [41] and *gar-2* reporters [40]; n = 30–80 male tail sides; “w” denotes weak marker expression. In the control RNAi experiments, *C. elegans* transgenic strains were fed a bacterial strain that synthesizes dsRNA for the pseudogene C06C3.5.
(PPT)

Table S2 **The DBL-1 Pathway blocks DMD-3 activity in rays 5, 7 and 9.** Relates to Fig. 3. See legend Table S1. n = 24 to 69 male tail sides scored.
(PPT)

## References

[1] Burtis K, Coschigano K, Baker B, Wensink P (1991). The doublesex proteins of *Drosophila melanogaster* bind directly to a sex-specific yolk protein gene enhancer.. EMBO J.

[2] Raymond CS, Shamu CE, Shen MM, Seifert KJ, Hirsch B (1998). Evidence for evolutionary conservation of sex-determining genes.. Nature.

[3] Raymond CS, Murphy MW, O'Sullivan MG, Bardwell VJ, Zarkower D (2000). Dmrt1, a gene related to worm and fly sexual regulators, is required for mammalian testis differentiation.. Genes Dev.

[4] Williams T, Carroll SB (2009). Genetic and molecular insights into the development and evolution of sexual dimorphism.. Nat Rev Genet.

[5] Kopp A, Duncan I, Carroll SB (2000). Genetic control and evolution of sexually dimorphic characters in *Drosophila*.. Nature.

[6] Yi W, Ross JM, Zarkower D (2000). *Mab-3* is a direct *tra-1* target gene regulating diverse aspects of *C. elegans* male sexual development and behavior.. Development.

[7] Estrada B, Sanchez-Herrero E (2001). The Hox gene *Abdominal-B* antagonizes appendage development in the genital disc of *Drosophila*.. Development.

[8] Keisman EL, Christiansen AE, Baker BS (2001). The sex determination gene *doublesex* regulates the A/P organizer to direct sex-specific patterns of growth in the *Drosophila* genital Iimaginal disc.. Dev Cell.

[9] Sanchez L, Gorfinkiel N, Guerrero I (2001). Sex determination genes control the development of the *Drosophila* genital disc, modulating the response to Hedgehog, Wingless and Decapentaplegic signals.. Development.

[10] Ross JM, Kalis AK, Murphy MW, Zarkower D (2005). The DM domain protein MAB-3 promotes sex-specific neurogenesis in *C. elegans* by regulating bHLH proteins.. Dev Cell.

[11] Williams TM, Selegue JE, Werner T, Gompel N, Kopp A (2008). The regulation and evolution of a genetic switch controlling sexually dimorphic traits in *Drosophila*.. Cell.

[12] Chatterjee SS, Uppendahl LD, Chowdhury MA, Ip P-L, Siegal ML (2011). The female-specific Doublesex isoform regulates pleiotropic transcription factors to pattern genital development in *Drosophila*.. Development.

[13] Lints R, Emmons SW (2002). Regulation of sex-specific differentiation and mating behavior in *C. elegans* by a new member of the DM domain transcription factor family.. Genes Dev.

[14] Balciuniene J, Bardwell VJ, Zarkower D (2006). Mice mutant in the DM domain gene *Dmrt4* are viable and fertile but have polyovular follicles.. Mol Cell Biol.

[15] Rideout EJ, Billeter J-C, Goodwin SF (2007). The sex-determination genes *fruitless* and *doublesex* specify a neural substrate required for courtship song.. Curr Biol.

[16] Rideout EJ, Dornan AJ, Neville MC, Eadie S, Goodwin SF (2010). Control of sexual differentiation and behavior by the *doublesex* gene in *Drosophila melanogaster*.. Nat Neurosci.

[17] Gennet N, Gale E, Nan X, Farley E, Takacs K (2011). Doublesex and mab-3–related transcription factor 5 promotes midbrain dopaminergic identity in pluripotent stem cells by enforcing a ventral-medial progenitor fate.. Proc Natl Acad Sci.

[18] Ward S, Thomson N, White J, Brenner S (1975). Electron microscopical reconstruction of the anterior sensory anatomy of the nematode *Caenorhabditis elegans*.. J Comp Neurol.

[19] Sulston J, Horvitz H (1977). Post-embryonic cell lineages of the nematode, *Caenorhabditis elegans*.. Dev Biol.

[20] Sulston J, Albertson D, Thomson J (1980). The *Caenorhabditis elegans* male: Postembryonic development of nongonadal structures.. Dev Biol.

[21] Sulston J, Schierrenberg E, White J, Thomson J (1983). The embryonic cell lineage of the nematode *Caenorhabditis elegans*.. Dev Biol.

[22] White J, Southgate E, Thomson J, Brenner S (1986). The structure of the nervous system of the nematode *Caenorhabditis elegans*.. Phil Trans Royal Soc London Series B, Biol Scien.

[23] Hall D, Russell R (1991). The posterior nervous system of the nematode *Caenorhabditis elegans*: Serial reconstruction of identified neurons and complete pattern of synaptic interactions.. J Neurosci.

[24] Portman DS, Daisuke Y (2007). Genetic control of sex differences in *C. elegans* neurobiology and behavior.. Advances in Genetics.

[25] Lee K, Portman DS (2007). Neural sex modifies the function of a *C. elegans* sensory circuit.. Curr Biol.

[26] Male Wiring Project.. http://worms.aecom.yu.edu/PHP/male_wiring_project.php.

[27] Mason DA, Rabinowitz JS, Portman DS (2008). *dmd-3*, a *doublesex*-related gene regulated by *tra-1*, governs sex-specific morphogenesis in *C. elegans*.. Development.

[28] Koo P, Bian X, Sherlekar A, Bunkers M, Lints R (2011). The robustness of *Caenorhabditis elegans* male mating behavior depends on the distributed properties of ray sensory neurons and their output through core and male-specific targets.. J Neurosci.

[29] Loer C, Kenyon C (1993). Serotonin-deficient mutants and male mating behavior in the nematode *Caenorhabditis elegans*.. J Neurosci.

[30] Lints R, Jia L, Kim K, Li C, Emmons SW (2004). Axial patterning of *C. elegans* male sensilla identities by selector genes.. Dev Biol.

[31] Flames N, Hobert O (2009). Gene regulatory logic of dopamine neuron differentiation.. Nature.

[32] Portman DS, Emmons SW (2004). Identification of *C. elegans* sensory ray genes using whole-genome expression profiling.. Dev Biol.

[33] Sulston J, Dew M, Brenner S (1975). Dopaminergic neurons in the nematode *Caenorhabditis elegans*.. J Comp Neurol.

[34] Jayanthi LD, Apparsundaram S, Malone MD, Ward E, Miller DM (1998). The *Caenorhabditis elegans* gene *T23G5.5* encodes an antidepressant- and cocaine-sensitive dopamine transporter.. Mol Pharmacol.

[35] Duerr JS, Frisby DL, Gaskin J, Duke A, Asermely K (1999). The *cat-1* gene of *Caenorhabditis elegans* encodes a vesicular monoamine transporter required for specific monoamine-dependent behaviors.. J Neurosci.

[36] Lints R, Emmons SW (1999). Patterning of dopaminergic neurotransmitter identity among *Caenorhabditis elegans* ray sensory neurons by a TGFbeta family signaling pathway and a Hox gene.. Development.

[37] Hare E, Loer C (2004). Function and evolution of the serotonin-synthetic *bas-1* gene and other aromatic amino acid decarboxylase genes in *Caenorhabditis*.. BMC Evol Biol.

[38] Nass R, Hahn MK, Jessen T, McDonald PW, Carvelli L (2005). A genetic screen in *Caenorhabditis elegans* for dopamine neuron insensitivity to 6-hydroxydopamine identifies dopamine transporter mutants impacting transporter biosynthesis and trafficking.. J Neurochem.

[39] Alfonso A, Grundahl K, Duerr J, Han H, Rand JB (1993). The *Caenorhabditis elegans unc-17* gene: A putative vesicular acetylcholine transporter.. Science.

[40] Lee Y-S, Park Y-S, Nam S, Suh S, Lee J (2000). Characterization of GAR-2, a novel G protein-linked acetylcholine receptor from *Caenorhabditis elegans*.. J Neurochem.

[41] Garcia LR, Mehta P, Sternberg PW (2001). Regulation of distinct muscle behaviors controls the *C. elegans* male's copulatory spicules during mating.. Cell.

[42] Baird S, Fitch D, Kassem I, Emmons S (1991). Pattern formation in the nematode epidermis: Determination of the arrangement of peripheral sense organs in the *C. elegans* male tail.. Development.

[43] Savage C, Das P, Finelli A, Townsend S, Sun C (1996). *Caenorhabditis elegans* genes *sma-2*, *sma-3*, and *sma-4* define a conserved family of transforming growth factor beta pathway components.. Proc Natl Acad Sci.

[44] Krishna S, Maduzia L, Padgett R (1999). Specificity of TGFbeta signaling is conferred by distinct type I receptors and their associated SMAD proteins in *Caenorhabditis elegans*.. Development.

[45] Morita K, Chow K, Ueno N (1999). Regulation of body length and male tail ray pattern formation of *Caenorhabditis elegans* by a member of TGF-beta family.. Development.

[46] Suzuki Y, Yandell M, Roy P, Krishna S, Savage-Dunn C (1999). A BMP homolog acts as a dose-dependent regulator of body size and male tail patterning in *Caenorhabditis elegans*.. Development.

[47] Liu KS, Sternberg PW (1995). Sensory regulation of male mating behavior in *Caenorhabditis elegans*.. Neuron.

[48] Nagel G, Brauner M, Liewald JF, Adeishvili N, Bamberg E (2005). Light activation of Channelrhodopsin-2 in excitable cells of *Caenorhabditis elegans* triggers rapid behavioral responses.. Curr Biol.

[49] Barr MM, Sternberg PW (1999). A polycystic kidney-disease gene homologue required for male mating behaviour in *C. elegans*.. Nature.

[50] Hurd DD, Miller RM, Nunez L, Portman DS (2010). Specific alpha- and beta-tubulin isotypes optimize the functions of sensory cilia in *Caenorhabditis elegans*.. Genetics.

[51] Whittaker A, Sternberg P (2009). Coordination of opposing sex-specific and core muscle groups regulates male tail posture during *Caenorhabditis elegans* male mating behavior.. BMC Biology.

[52] Liu Y, LeBeouf B, Guo X, Correa PA, Gualberto DG (2011). A cholinergic-regulated circuit coordinates the maintenance and bi-stable states of a sensory-motor behavior during *Caenorhabditis elegans* male copulation.. PLoS Genet.

[53] Carnell L, Illi J, Hong SW, McIntire SL (2005). The G-protein-coupled serotonin receptor SER-1 regulates egg laying and male mating behaviors in *Caenorhabditis elegans*.. J Neurosci.

[54] Shirangi TR, Dufour HD, Williams TM, Carroll SB (2009). Rapid evolution of sex pheromone-producing enzyme expression in *Drosophila*.. PLoS Biol.

[55] Yang Y, Sun Y, Luo X, Zhang Y, Chen Y (2007). Polycomb-like genes are necessary for specification of dopaminergic and serotonergic neurons in *Caenorhabditis elegans*.. Proc Natl Acad Sci.

[56] Doitsidou M, Flames N, Lee AC, Boyanov A, Hobert O (2008). Automated screening for mutants affecting dopaminergic-neuron specification in *C. elegans*.. Nat Meth.

[57] Yi W, Zarkower D (1999). Similarity of DNA binding and transcriptional regulation by *Caenorhabditis elegans* MAB-3 and *Drosophila melanogaster* DSX suggests conservation of sex determining mechanisms.. Development.

[58] Murphy MW, Zarkower D, Bardwell V (2007). Vertebrate DM domain proteins bind similar DNA sequences and can heterodimerize on DNA.. BMC Mol Biol.

[59] Murphy MW, Sarver AL, Rice D, Hatzi K, Ye K (2010). Genome-wide analysis of DNA binding and transcriptional regulation by the mammalian Doublesex homolog DMRT1 in the juvenile testis.. Proc Natl Acad Sci.

[60] Yu JY, Kanai MI, Demir E, Jefferis GSXE, Dickson BJ (2010). Cellular organization of the neural circuit that drives *Drosophila* courtship behavior.. Curr Biol.

[61] Kohatsu S, Koganezawa M, Yamamoto D (2011). Female contact activates male-specific interneurons that trigger stereotypic courtship behavior in *Drosophila*.. Neuron.

[62] Sanders LE, Arbeitman MN (2008). Doublesex establishes sexual dimorphism in the Drosophila central nervous system in an isoform-dependent manner by directing cell number.. Developmental Biology.

[63] Bocklandt S, Vilain E, Daisuke Y (2007). Sex differences in brain and behavior: Hormones versus genes.. Adv Genet.

[64] Dewing P, Shi T, Horvath S, Vilain E (2003). Sexually dimorphic gene expression in mouse brain precedes gonadal differentiation.. Brain Res Mol Brain Res.

[65] Arnold AP, Burgoyne PS (2004). Are XX and XY brain cells intrinsically different?. Trends Endocrinol Metab.

[66] Dewing P, Chiang CWK, Sinchak K, Sim H, Fernagut P-O (2006). Direct regulation of adult brain function by the male-specific factor SRY.. Curr Biol.

[67] Guo Y, Li Q, Gao S, Zhou X, He Y (2004). Molecular cloning, characterization, and expression in brain and gonad of *Dmrt5* of zebrafish.. Biochem Biophys Res Commun.

[68] Huang X, Hong C-S, O'Donnell M, Saint-Jeannet J-P (2005). The doublesex-related gene, *XDmrt4*, is required for neurogenesis in the olfactory system.. Proc Natl Acad Sci U S A.

[69] Davies AG, Pierce-Shimomura JT, Kim H, VanHoven MK, Thiele TR (2003). A central role of the BK potassium channel in behavioral responses to ethanol in *C. elegans*.. Cell.

[70] Schmid C, Schwarz V, Hutter H (2006). AST-1, a novel ETS-box transcription factor, controls axon guidance and pharynx development in *C. elegans*.. Dev Biol.

[71] Hutter H, Wacker I, Schmid C, Hedgecock EM (2005). Novel genes controlling ventral cord asymmetry and navigation of pioneer axons in *C. elegans*.. Dev Biol.

[72] Brenner S (1974). The genetics of *Caenorhabditis elegans*.. Genetics.

[73] Granato M, Schnabel H, Schnabel R (1994). Genesis of an organ: Molecular analysis of the *pha-1* gene.. Development.

[74] Ferguson EL, Horvitz HR (1985). Identification and characterization of 22 genes that affect the vulval cell lineages of the nematode *Caenorhabditis elegans*.. Genetics.

[75] Hodgkin J, Horvitz HR, Brenner S (1979). Nondisjunction mutants of the nematode *Caenorhabditis elegans*.. Genetics.

[76] Ahringer J Reverse genetics (April 6, 2006), *WormBook*, ed.. http://www.wormbook.org.

[77] Rual J, Ceron J, Kooreth J, Hao T, Nicot A (2004). Toward improving *Caenorhabditis elegans* phenome mapping with an ORFeome-based RNAi library.. Genome Res.

[78] Wang D, Ruvkun G (2004). Regulation of *Caenorhabditis elegans* RNA interference by the *daf-2* insulin stress and longevity signaling pathway.. Cold Spring Harb Symp Quant Biol.

[79] Bargmann CI, Avery L (1995). Laser killing of cells in *Caenorhabditis elegans*. Methods Cell Biol.

[80] Liu Y, LeBoeuf B, Garcia LR (2007). G alpha(q)-coupled muscarinic acetylcholine receptors enhance nicotinic acetylcholine receptor signaling in *Caenorhabditis elegans* mating behavior.. J Neurosci.

