# Supplementary figures and images for: Multiple doublesex-Related Genes Specify Critical Cell Fates in a C. elegans Male Neural Circuit
Source: PLoS One. 2011 Nov 1;6(11):e26811. doi: 10.1371/journal.pone.0026811 (PMC3206049; doi:10.1371/journal.pone.0026811)

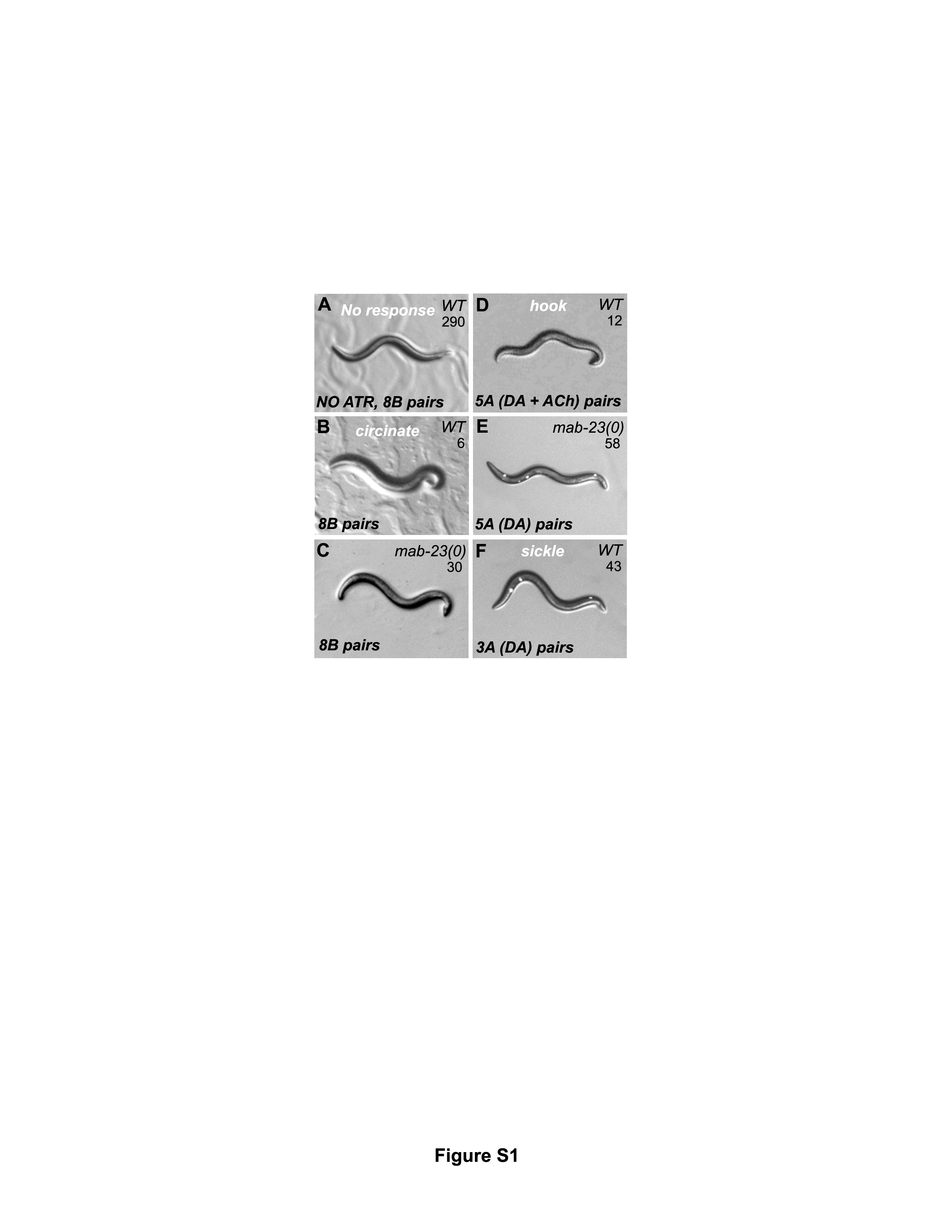

Supplement: Figure S1 — Ray neuron-induced tail-curl postures are abnormal in mab-23 mutants. Relates to Fig. 5. Images of tail postures generated by ChR2-YFP transgenic males after ChR2 activation with a blue light pulse (anterior left, posterior right). The number of ray neurons activated, their type (A or B) and neurotransmitter fate (DA or ACh for the A-neurons) are indicated. The Tail Curve value for each male posture is indicated in the upper right of each image (see Materials and Methods for derivation). Except for A, all males shown were grown in the presence of ChR2 co-factor all-trans-retinal (ATR). “Circinate”, “sickle” and “hook” refer to the shape of the tail posture. A. A wild type (WT) transgenic male grown without ATR (NO ATR) shows no change in tail posture when pulsed with blue light because ChR2 is non-functional in the absence of ATR. The transgenes used were as follows: A–C. ppkd-2::ChR2-YFP. D. ptba-9::ChR2-YFP. E, F. pdat-1::ChR2-YFP. The bright fluorescent spots in E and F correspond to expression of co-transformation marker UNC-122::GFP in coelomocytes. (TIF) [file pone.0026811.s001.tif]

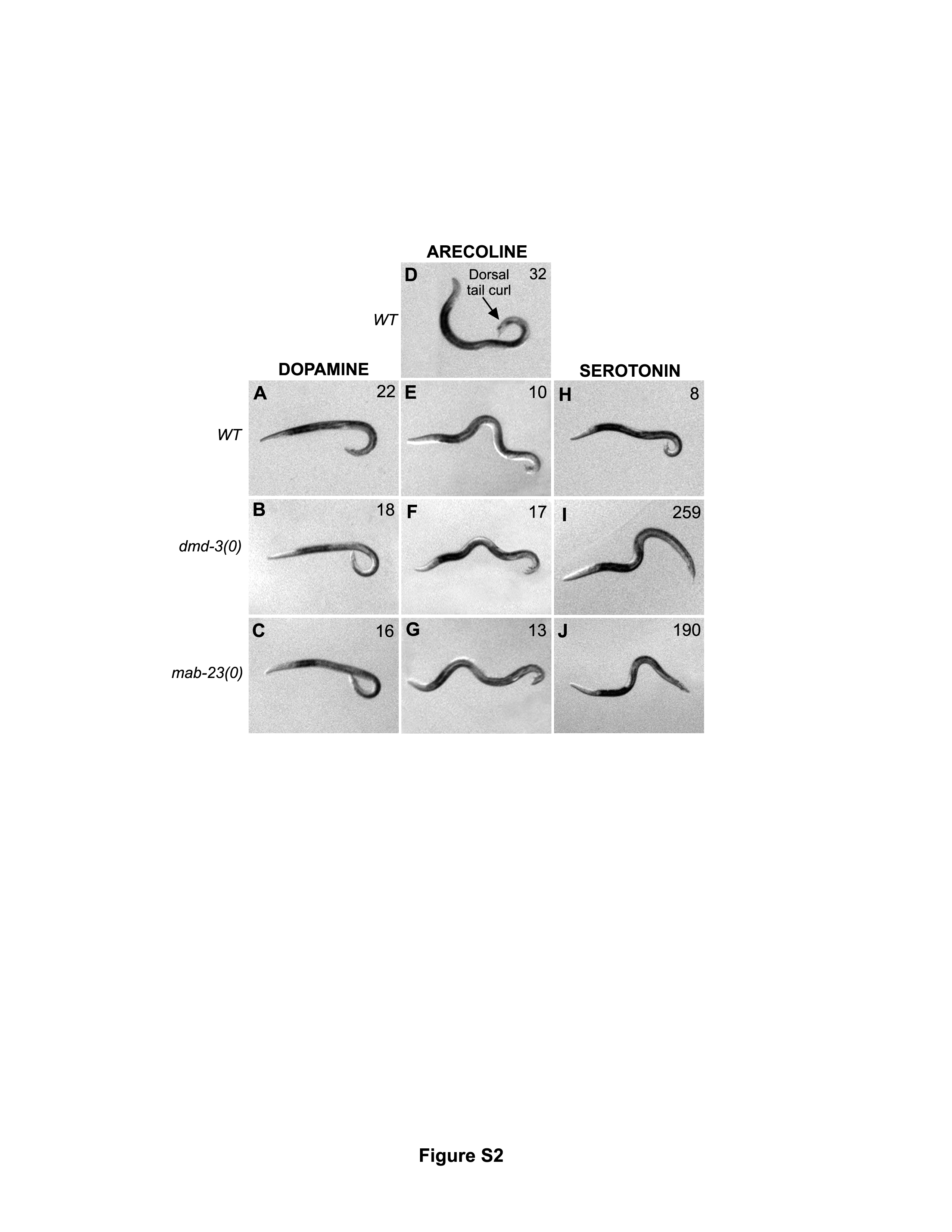

Supplement: Figure S2 — DM domain gene mutants show abnormal responses to exogenous neurotransmitter receptor agonists. Relates to Fig. 6. Images of wild type (WT), dmd-3(0) and mab-23(0) males after exposure to the neurotransmitter receptor agonist indicated (anterior left and posterior right, see Materials and Methods). Tail Curve values (top right) were derived as described for ChR2 assays. D shows a WT type male curling its tail dorsally when exposed to arecoline. dmd-3 and mab-23 males rarely curl their tails dorsally in this agonist. (TIF) [file pone.0026811.s002.tif]

## Slide 1
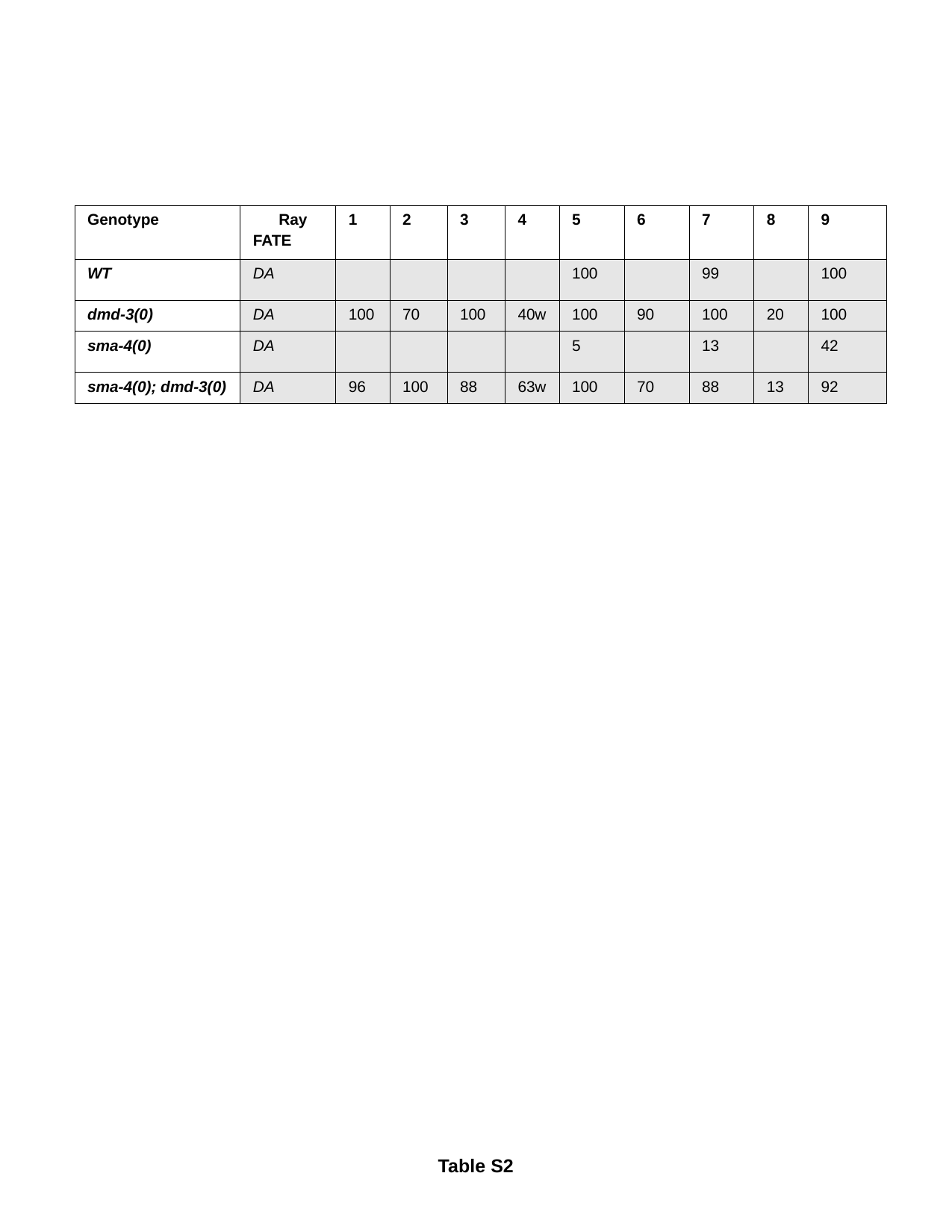

| Genotype | Ray FATE | 1 | 2 | 3 | 4 | 5 | 6 | 7 | 8 | 9 |
| --- | --- | --- | --- | --- | --- | --- | --- | --- | --- | --- |
| WT | DA | | | | | 100 | | 99 | | 100 |
| dmd-3(0) | DA | 100 | 70 | 100 | 40w | 100 | 90 | 100 | 20 | 100 |
| sma-4(0) | DA | | | | | 5 | | 13 | | 42 |
| sma-4(0); dmd-3(0) | DA | 96 | 100 | 88 | 63w | 100 | 70 | 88 | 13 | 92 |
Table S2

Supplement: Table S2 — The DBL-1 Pathway blocks DMD-3 activity in rays 5, 7 and 9. Relates to Fig. 3. See legend Table S1. n = 24 to 69 male tail sides scored. (PPT) [file pone.0026811.s004.ppt]
