# Supplementary material for: Multiple doublesex-Related Genes Specify Critical Cell Fates in a C. elegans Male Neural Circuit
Source: PLoS One. 2011 Nov 1;6(11):e26811. doi: 10.1371/journal.pone.0026811 (PMC3206049; doi:10.1371/journal.pone.0026811)
Supplement: Table S1 — dmd-3 , mab-23 and ast-1 regulate DA/ACh fate choice in the ray A-neurons. Relates to Fig. 3. Expression of A-neuron fates in the genetic backgrounds indicated. The percentage of males that express the marker indicated in a particular ray is shown. Typically one side per male was scored. Dopaminergic (DA) fate: cat-2 or dat-1 reporters [36], [69], cholinergic (ACh) fate: unc-17 [41] and gar-2 reporters [40]; n = 30–80 male tail sides; “w” denotes weak marker expression. In the control RNAi experiments, C. elegans transgenic strains were fed a bacterial strain that synthesizes dsRNA for the pseudogene C06C3.5. (PPT) [file pone.0026811.s003.ppt]

## Slide 1
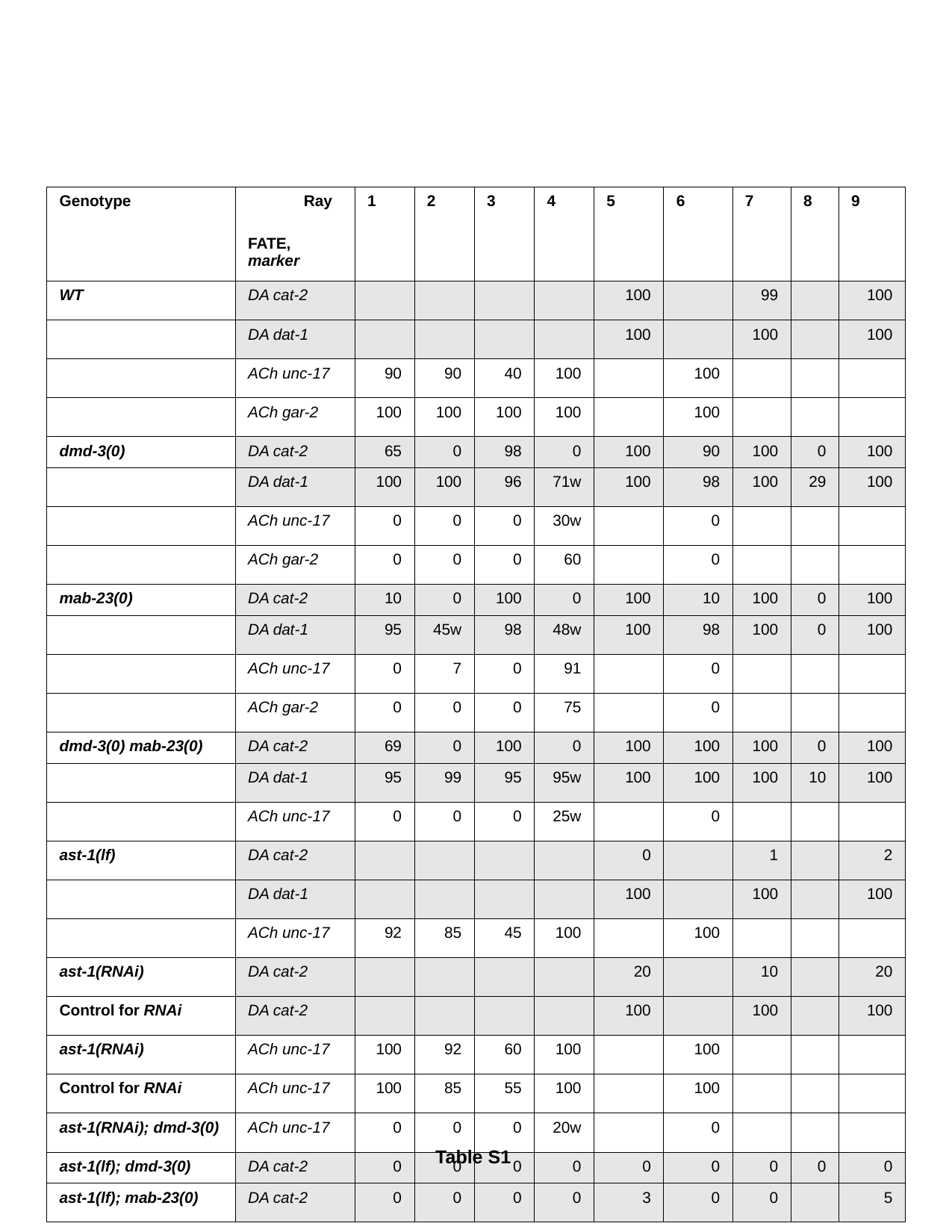

| Genotype | Ray FATE, marker | 1 | 2 | 3 | 4 | 5 | 6 | 7 | 8 | 9 |
| --- | --- | --- | --- | --- | --- | --- | --- | --- | --- | --- |
| WT | DA cat-2 | | | | | 100 | | 99 | | 100 |
| | DA dat-1 | | | | | 100 | | 100 | | 100 |
| | ACh unc-17 | 90 | 90 | 40 | 100 | | 100 | | | |
| | ACh gar-2 | 100 | 100 | 100 | 100 | | 100 | | | |
| dmd-3(0) | DA cat-2 | 65 | 0 | 98 | 0 | 100 | 90 | 100 | 0 | 100 |
| | DA dat-1 | 100 | 100 | 96 | 71w | 100 | 98 | 100 | 29 | 100 |
| | ACh unc-17 | 0 | 0 | 0 | 30w | | 0 | | | |
| | ACh gar-2 | 0 | 0 | 0 | 60 | | 0 | | | |
| mab-23(0) | DA cat-2 | 10 | 0 | 100 | 0 | 100 | 10 | 100 | 0 | 100 |
| | DA dat-1 | 95 | 45w | 98 | 48w | 100 | 98 | 100 | 0 | 100 |
| | ACh unc-17 | 0 | 7 | 0 | 91 | | 0 | | | |
| | ACh gar-2 | 0 | 0 | 0 | 75 | | 0 | | | |
| dmd-3(0) mab-23(0) | DA cat-2 | 69 | 0 | 100 | 0 | 100 | 100 | 100 | 0 | 100 |
| | DA dat-1 | 95 | 99 | 95 | 95w | 100 | 100 | 100 | 10 | 100 |
| | ACh unc-17 | 0 | 0 | 0 | 25w | | 0 | | | |
| ast-1(lf) | DA cat-2 | | | | | 0 | | 1 | | 2 |
| | DA dat-1 | | | | | 100 | | 100 | | 100 |
| | ACh unc-17 | 92 | 85 | 45 | 100 | | 100 | | | |
| ast-1(RNAi) | DA cat-2 | | | | | 20 | | 10 | | 20 |
| Control for RNAi | DA cat-2 | | | | | 100 | | 100 | | 100 |
| ast-1(RNAi) | ACh unc-17 | 100 | 92 | 60 | 100 | | 100 | | | |
| Control for RNAi | ACh unc-17 | 100 | 85 | 55 | 100 | | 100 | | | |
| ast-1(RNAi); dmd-3(0) | ACh unc-17 | 0 | 0 | 0 | 20w | | 0 | | | |
| ast-1(lf); dmd-3(0) | DA cat-2 | 0 | 0 | 0 | 0 | 0 | 0 | 0 | 0 | 0 |
| ast-1(lf); mab-23(0) | DA cat-2 | 0 | 0 | 0 | 0 | 3 | 0 | 0 | | 5 |
Table S1
